# A Multi-Method Approach to Analyzing MOFs for Chemical Warfare Simulant Capture: Molecular Simulation, Machine Learning, and Molecular Fingerprints

**DOI:** 10.3390/nano15030183

**Published:** 2025-01-24

**Authors:** Zhongyuan Ming, Min Zhang, Shouxin Zhang, Xiaopeng Li, Xiaoshan Yan, Kexin Guan, Yu Li, Yufeng Peng, Jinfeng Li, Heguo Li, Yue Zhao, Zhiwei Qiao

**Affiliations:** 1 State Key Laboratory of NBC Protection for Civilian, Institute of Chemical Defense, Beijing 100191, China; mingzhongyuan2022@163.com (Z.M.); minjohng@126.com (M.Z.); zhangsxfhyjy@163.com (S.Z.); lxpbuct@163.com (X.L.); yanxiaoshan07@foxmail.com (X.Y.); 2 Guangzhou Key Laboratory for New Energy and Green Catalysis, School of Chemistry and Chemical Engineering, Guangzhou University, Guangzhou 510006, China; 2112105072@e.gzhu.edu.cn (K.G.); liyu2021@e.gzhu.edu.cn (Y.L.); yfpeng@icost.ac.cn (Y.P.); 2112205096@e.gzhu.edu.cn (J.L.)

**Keywords:** chemical warfare agents, metal-organic frameworks, high-throughput computational screening, interpretable machine learning, molecular fingerprint

## Abstract

Mustard gas (HD) is a well-known chemical warfare agent, recognized for its extreme toxicity and severe hazards. Metal–organic frameworks (MOFs), with their unique structural properties, show significant potential for HD adsorption applications. Due to the extreme hazards of HD, most experimental studies focus on its simulants, but molecular simulation research on these simulants remains limited. Simulation analyses of simulants can uncover structure–performance relationships and enable experimental validation, optimizing methods, and improving material design and performance predictions. This study integrates molecular simulations, machine learning (ML), and molecular fingerprinting (MFs) to identify MOFs with high adsorption performance for the HD simulant diethyl sulfide (DES), followed by in-depth structural analysis and comparison. First, MOFs are categorized into Top, Middle, and Bottom materials based on their adsorption efficiency. Univariate analysis, machine learning, and molecular fingerprinting are then used to identify and compare the distinguishing features and fingerprints of each category. Univariate analysis helps identify the optimal structural ranges of Top and Bottom materials, providing a reference for initial material screening. Machine learning feature importance analysis, combined with SHAP methods, identifies the key features that most significantly influence model predictions across categories, offering valuable insights for future material design. Molecular fingerprint analysis reveals critical fingerprint combinations, showing that adsorption performance is optimized when features such as metal oxides, nitrogen-containing heterocycles, six-membered rings, and C=C double bonds co-exist. The integrated analysis using HTCS, ML, and MFs provides new perspectives for designing high-performance MOFs and demonstrates significant potential for developing materials for the adsorption of CWAs and their simulants.

## 1. Introduction

Bis(2-chloroethyl) sulfide, commonly known as mustard gas (HD), is a representative chemical warfare agent (CWA). HD can penetrate the human body through the skin, respiratory tract, and eyes, causing severe tissue damage and, in some cases, resulting in blindness or death. Due to its high-risk properties, HD has become one of the primary subjects of CWA research [1]. However, direct experimental studies on HD’s hazards and decontamination mechanisms carry significant risks. As a result, researchers commonly use simulants with lower toxicity and similar properties to study HD’s adsorption behavior indirectly. Diethylsulfide (DES), which shares a similar structure with HD but is significantly less toxic, is widely used as an HD simulant [2,3]. DES can accurately simulate HD’s adsorption behavior, offering valuable insights for developing high-performance adsorption materials [4,5,6,7]. Currently, porous materials such as activated carbon and metal oxides are widely used for CWA adsorption. However, their uneven pore size distribution and limited chemical tunability result in performance instability, thereby limiting their practical effectiveness [8,9].

Recent studies increasingly indicate that metal–organic frameworks (MOFs) offer substantial advantages over traditional materials in CWA adsorption [10]. MOFs, constructed from metal clusters and organic linkers, exhibit exceptionally high surface areas, diverse pore structures, and tunable chemical properties, which facilitate efficient molecular capture and selective adsorption [11,12,13]. Therefore, MOFs hold significant potential for the development of CWA adsorbents [10,14,15]. However, research on the interactions between MOFs and DES has primarily concentrated on sensor materials. Samokhvalov et al. [16] identified the main adsorption sites for DES on MOFs with zinc porphyrin aluminum as the porphyrin ring and carboxyl groups, revealing the interactions between the functional groups and DES. Additionally, they also investigated the dynamic interactions between MOFs and DES vapor and explored the impact of water vapor on the DES adsorption performance of MOFs [17].

However, the experimental synthesis and screening of suitable MOF adsorbents are time-consuming, labor-intensive, and costly, significantly limiting their large-scale application. Thus, employing high-throughput molecular simulation techniques to preliminarily assess the adsorption performance of MOFs before experimentation can aid in screening potential materials, reducing experimental steps, conserving resources, and accelerating research progress [18,19]. The uniform and periodic structure of MOFs provides significant advantages for integrating computational and experimental research [20]. High-throughput computational screening allows for the rapid prediction of MOF adsorption performance in databases containing large numbers of material samples [21]. This approach not only reveals structure–performance relationships but also efficiently identifies high-performance materials, enabling experimental efforts to focus on the most promising candidates [21,22,23]. For example, Agrawal et al. simulated the adsorption enthalpies of over 2900 MOFs for Sarin and Soman, identifying dimethyl methylphosphonate (DMNP) as the optimal simulant for Soman and dimethyl methylphosphonate (DMMP) as the best simulant for Sarin. Additionally, DMMP and dicumyl peroxide (DCP) were also identified as close structural analogs of Sarin [24]. Wang et al. investigated the adsorption mechanisms of DMMP on zirconium-based MOFs, providing additional insights into the critical interactions between MOFs and chemical warfare agents [25]. Research by Maurin et al. showed that a series of water-stable zirconium- and titanium-based MOFs exhibited excellent adsorption performance for Soman, Sarin, and their simulants [26]. Samokhvalov et al. [27] employed density functional theory (DFT) to simulate the interactions between diethyl sulfide (DES) and aluminum-based porphyrin metal–organic frameworks (Al-MOFs), revealing how DES molecules interact with the metal centers within the framework and functional groups (such as µ(O–H) and COO−) on the organic linkers, which could be critical for sensor applications. Although molecular simulations are more efficient and cost-effective than experiments, they still require substantial computational resources and are limited by computational capacity and methodological constraints [28].

With the advent of the big data era, machine learning (ML) has found widespread application in materials science, leveraging its data-driven nature [29,30,31]. Unlike traditional calculation methods based on physical equations, ML analyzes patterns in large datasets to rapidly predict material properties, significantly reducing computational costs [29]. Machine learning can be applied not only to the prediction and optimization of materials, but also to reverse engineering and process optimization, thereby driving innovation across the entire material design process [32].

ML models based on the structural features of MOFs have been successfully used to screen various MOF materials and identify optimal adsorbents [33,34]. Puzari et al. [35] provided a summary of molecular simulation techniques and their integration with machine learning (ML) for predicting MOF crystal structures and mechanical properties. The study underscores the critical role of AI in the design and discovery of MOF materials, particularly through its integration with molecular simulations to enhance both efficiency and accuracy. For classification tasks, ML models can also efficiently categorize MOFs [36,37,38]. ML-assisted high-throughput computational screening (HTCS) strategies have received significant attention [29,39]. For instance, Bucior et al. developed a regression model to predict the hydrogen adsorption performance of MOFs [40]. Yang et al. [41] applied four ML models to accurately predict the adsorption performance of hydrophobic MOFs and identified, through ML analysis, that void fraction (*ϕ*) and adsorption enthalpy are key factors influencing performance prediction. In molecular simulations of DMMP, 2-CEES, Soman, and HD, Wang et al. [42] found that the number of hydrogen bond acceptors is a critical factor controlling the co-adsorption of CWAs and their simulants. To further explore structure–performance relationships, many studies have combined ML with molecular fingerprints (MFs) to more efficiently screen high-performance MOFs. For example, Lin et al. [43] used molecular fingerprinting methods, including Morgan and MACCS, to decode the complex organic linkers in MOFs, incorporating secondary building units (SBUs) and topological structures to construct predictive ML models, thereby offering guidance for MOF design. Yuan et al. [44] combined machine learning models with four molecular fingerprinting techniques and found that the MACCS fingerprint outperformed the others. Ni et al. [45] integrated 166-bit MACCS molecular fingerprints into an ML model to study the influence of functional groups on HD adsorption performance, identifying 22 key functional groups through the analysis of numerous high-performance MOFs. These studies highlight the crucial role of ML and MFs in facilitating high-throughput computational screening, accelerating the adsorbent selection process and providing atomic-level insights for structural design.

Currently, simulation studies of CWAs primarily focus on HD and CEES, whereas research on DES remains relatively limited. Matito-Martos et al. [46] were the first to incorporate DES into molecular simulations. However, systematic studies on the adsorption of DES by MOFs, incorporating ML and MFs, remain scarce. While most studies focus on CWAs, simulants have received comparatively less attention. Molecular simulations and big data mining of simulants not only provide guidance for subsequent experiments but also facilitate the experimental validation of the simulations’ accuracy.

Therefore, this study integrates molecular simulation, ML, and MF techniques to identify MOFs with high adsorption performance for DES and to explore their structure–performance relationships in depth. First, univariate analysis was performed to investigate the influence of different descriptors on adsorption performance. Based on the TSN metric, the CoRE-MOFs database was divided into three categories (Top, Middle, and Bottom), with the structural feature ranges for each category clearly defined. Next, four ML regression models (LGBM, XGB, CatBoost, and RF) were applied in conjunction with the interpretable Shapley Additive Explanation (SHAP) algorithm to reveal the key differences among material categories. To assess the transferability and scalability of the ML models, the LGBM model was further validated on the hMOFs database. Using the MACCS molecular fingerprinting algorithm and two statistical methods, the relative proportions of MFs across Top, Middle, and Bottom materials were calculated, identifying key structural components specific to Top materials through comparisons with Bottom materials. The combined analysis of HTCS, ML, and MFs offers an innovative and effective approach for researching high-performance MOFs for DES adsorption.

## 2. Models and Methods

### 2.1. MOF Databases and Molecular Models

The MOF models used in this study are sourced from the 2019 CoRE-MOF database established by Chuang et al. [47] and the hMOF database [48,49]. The CoRE-MOF descriptors are categorized into four categories: structural, energetic, chemical, and charge descriptors. Structural descriptors include six parameters: largest cavity diameter (LCD, Å), pore limiting diameter (PLD, Å), porosity (*ϕ* or HVF), volumetric surface area (VSA, m^2^/cm^3^), and density (*ρ*, kg/m^3^). The largest cavity diameter and pore limiting diameter were calculated using the Zeo++-0.3 software [50], while *ϕ* and the VSA were obtained with the RASPA 2.0.45 software package [51], using helium (2.58 Å) and nitrogen (3.64 Å) as probe molecules. Additionally, the NVT-MC method in RASPA (N for particle number, V for system volume, T for system temperature, and MC for Monte Carlo) was used under infinite dilution conditions to calculate the adsorption energy of various MOFs, yielding adsorption heat (kJ/mol) and Henry’s coefficient (KH, mol/(kg⋅Pa)), which are included as energetic descriptors. Chemical descriptors primarily include metal content, percentages of carbon, hydrogen, oxygen, and nitrogen, total unsaturation, and unsaturation per carbon atom. Charge descriptors include the most positive charge (MPC) and average metal charge (AMC). Force field parameters for DES, N_2_, and O_2_ are derived from the TraPPE force field [46,52]. Detailed information on these descriptors, the DES model, and the force field parameters is provided in Tables S1–S4.

### 2.2. Simulation Details

The Grand Canonical Monte Carlo (GCMC) method is employed to simulate the adsorption and separation performance of CoRE-MOFs and hMOFs for various adsorbates. The non-bonding intermolecular interactions between MOFs and adsorbates primarily consist of two types: short-range van der Waals interactions and long-range electrostatic interactions. The sum of these two interactions (U_LJ+elec(r)_) represents the total energy:

(1) $$U_{LJ+elec}\left(r\right)=\sum 4\varepsilon_{ij}\left[{\left(\frac{\sigma_{ij}}{r_{ij}}\right)}^{12}-{\left(\frac{\sigma_{ij}}{r_{ij}}\right)}^{6}\right]+\sum \frac{q_{i}q_{j}}{4\pi \varepsilon_{0}r_{ij}}$$

In Equation (1), U_LJ+elec(r)_ represents the non-bonded interaction energy between atoms i and j, where the first term corresponds to the van der Waals non-bonding potential energy, and the second term corresponds to the Coulombic electrostatic potential energy. Here, q_i_ is the charge of atom i, ε_0_ = 8.8542 × 10^−12^ C^2^/N·m^2^ is the vacuum permittivity constant, r_ij_ is the distance between atoms i and j, ε_ij_ is the energy difference from the minimum energy point to zero energy, and σ_ij_ is the collision diameter. Atomic charges in MOFs were calculated using the EQeq method [53]. The LJ potential parameters for all CoRE-MOFs were derived from the Universal Force Field (UFF), the accuracy of which has been validated by multiple studies [54,55,56] (see Supporting Information for details). In the simulations, MOF structures are assumed to be rigid, with the atomic positions of the MOFs held fixed and all free solvents in the crystals removed, which is a common assumption when handling large numbers of adsorbent structures. Additionally, the bond angle bending potential, dihedral angle bending potential, and bond length stretching potential of MOFs were not considered, thereby neglecting intramolecular bonding interactions between MOF atoms.

In this study, the capture performance of CoRE-MOFs for trace DES (1000 ppm) in air (N_2_:O_2_ = 78:22) was evaluated at 298 K and 101.325 kPa, and the adsorption behavior of DES under these conditions was explored. The infinite dilution method provided critical insights into the interactions between DES and the adsorbent at low concentrations. The GCMC simulations for each MOF were performed independently, with the chemical potential, volume, and temperature held constant throughout the simulations. Interactions between MOFs and adsorbates were modeled using the Lorentz–Berthelot mixing rule, while interactions between adsorbates and framework atoms were described by the Lennard–Jones (LJ) potential, with a spherical cutoff radius of 12 Å and long-range corrections. Electrostatic interactions were calculated using the Ewald method with a cutoff radius of 12 Å. Each simulation point consisted of 100,000 Monte Carlo cycles, with the first 50,000 cycles used to equilibrate the system and the remaining 50,000 cycles used to compute cumulative averages. All simulations were performed using the RASPA software package [51]. During each cycle, translation, rotation, swap, regeneration, insertion, and deletion moves were carried out to achieve system equilibrium.

### 2.3. Machine Learning

To further investigate the structure–performance relationship between diethyl sulfide (DES) and CoRE-MOFs, this study employs four machine learning regression algorithms—CatBoost, LightGBM, XGBoost, and Random Forest—based on 16 feature variables, evaluated using three performance metrics. Further details on the machine learning algorithms are provided in Figures S1–S4, with specifics on hyperparameter optimization found in Figures S5 and S6 and Table S5. To ensure reproducibility, a fixed seed was used for the random number generator. For each machine learning algorithm, the dataset was randomly split into training and testing sets, with 70% allocated for model training and 30% for testing. Five-fold cross-validation was applied and repeated five times to ensure model stability, with the final results reported as the average of the cross-validation runs. Model accuracy was evaluated using R^2^, MAE, and RMSE metrics; further details are provided in the Supporting Information (SI), Sections S2–S4.

Although machine learning models can accurately predict MOF adsorption performance, an excessively large number of trees or neurons may lead to overfitting, making the model difficult to interpret. Therefore, an interpretable algorithm was employed to analyze feature importance and identify the positive or negative impact of each feature on the model. SHAP, a widely used method, explains model predictions by quantifying the marginal contribution of each input feature [57]. Detailed information on the SHAP algorithm is provided in the Supplementary Materials.

To further analyze the transferability of the optimal machine learning model and comprehensively assess its generalization ability and stability, an uncertainty analysis method was employed. The core idea of this method is to evaluate model prediction uncertainty by making multiple predictions on the same dataset. Specifically, the model underwent multiple iterative predictions on the test set, with the standard deviation of each set of predictions calculated as an uncertainty metric. This approach is similar to Monte Carlo Dropout but does not involve the Dropout mechanism used in neural networks, making it more suitable for tree-based models like LightGBM and XGBoost. In the uncertainty analysis, 100 iterations were performed, with each iteration predicting on the same test set. The standard deviation of these predictions was then calculated to quantify the model uncertainty. This method effectively identifies model adaptability and transferability to new datasets, offering deeper insights into model performance; further details are provided in the Supplementary Materials.

### 2.4. Molecular Fingerprint

To explore the design principles of high-performance MOFs, this study used the OpenBabel [58] and PaDEL-Descriptor [59] software packages to compute Molecular ACCess Systems (MACCS) fingerprints [60] of CoRE-MOFs. This enabled the analysis of their functional groups, bonding patterns, and other structural components. OpenBabel is an open-source chemistry toolbox used to visualize chemical data and convert between various chemical structure file formats. CIF files were converted to SDF format, which is the preferred input for PaDEL-Descriptor, a widely used tool for calculating molecular descriptors and fingerprints. MACCS fingerprints are available in two keysets: 960-bit and 166-bit, with the 166-bit keyset widely used for its simplicity and practicality. More details on molecular fingerprints can be found in Table S8. Furthermore, the MOFid scheme was used to characterize the building blocks of MOFs, including metal centers and topological structures, with a focus on metal analysis [61]. Each metal element was encoded as a separate binary feature using one-hot encoding, as shown in Figure S1. These feature columns were then used in subsequent analyses to assess the impact of metal elements on MOF performance. This encoding method effectively captures the diversity of metal types and provides a rich set of input features for the model.

## 3. Results and Discussion

### 3.1. Univariate Analysis

**Figure 1:**
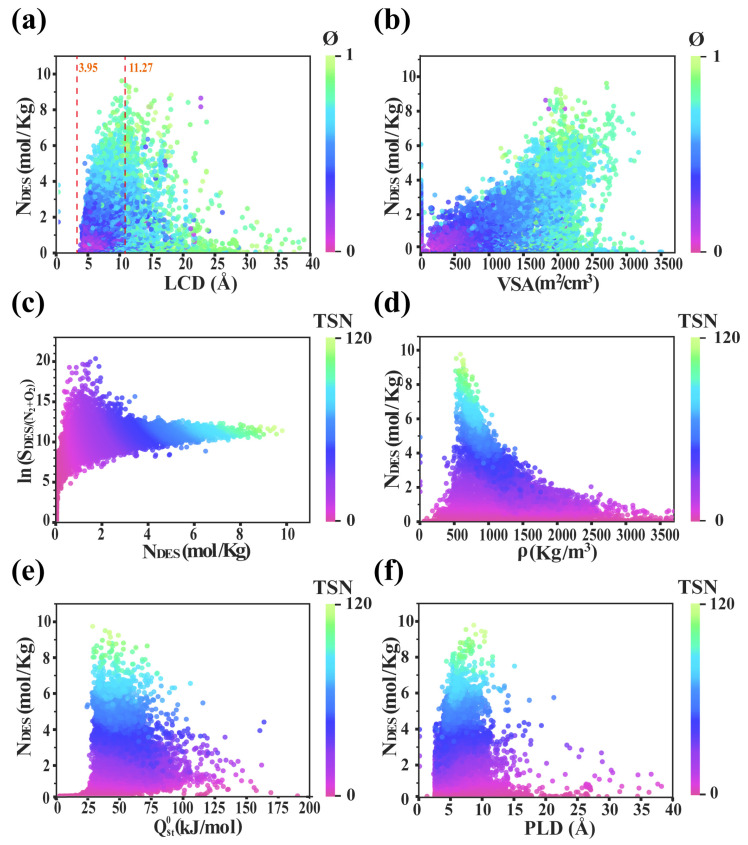
Descriptors–performance relationships: (**a**) LCD, *N_DES_*, and *ϕ*; (**b**) VSA, *N_DES_*, and *ϕ*; (**c**) *N_DES_*, lnS, and TSN; (**d**) *ρ*, *N_DES_*, and TSN; (**e**) $Q_{st}^{0}$, *N_DES_*, and *ϕ*; (**f**) PLD, *N_DES_*, and TSN.

High-throughput computational screening was initially performed on the DES adsorption performance (*N_DES_*) of CoRE-MOF materials, focusing on analyzing the influence of structural descriptors (LCD, PLD, and VSA) and energy descriptors $Q_{st}^{0}$ on *N_DES_*. Preliminary screening of *ϕ* is particularly crucial in univariate analysis. Previous studies have shown that among the top 1000 CoRE-MOFs for HD and its simulant CEES adsorption performance, *ϕ* is the most influential factor affecting adsorption efficiency [42]. In Figure 1, *ϕ* serves as a color map to highlight the relationships between each structural descriptor and adsorption performance.

Figure 1a illustrates the structure–performance relationship between *N_DES_* and LCD, where the overall trend initially increases and then decreases. When LCD is less than 3.95 Å, spatial limitations restrict DES adsorption in MOFs, preventing DES molecules from entering the pores, leading to almost zero adsorption [62]. Within the range of 3.95 to 11.27 Å, as LCD increases, the contact area between DES molecules and the MOF pore walls expands, which enhances intermolecular interactions and leads to a significant increase in adsorption. However, as LCD further increases, more N_2_ and O_2_ molecules enter the pores, leading to a decline in both selectivity and adsorption. When LCD exceeds 11.27 Å, *N_DES_* drops sharply, as larger pore diameters reduce contact between DES and the pore walls, weakening adsorption forces and significantly lowering adsorption capacity, often approaching zero. As *ϕ* increases, *N_DES_* gradually rises, indicating effective utilization of the MOF’s pore structure within an optimal porosity range [63]. However, as porosity continues to increase, *N_DES_* begins to decline. This is attributed to the introduction of larger pores at high porosity, which inefficiently utilize the pore space and reduce *N_DES_*. Figure 1b shows that as the VSA increases, *N_DES_* initially rises and then declines rapidly. When the VSA is low (0–1000 m^2^/cm^3^), *N_DES_* is also low, indicating a limited number of adsorption sites and insufficient adsorption capacity in MOFs. As the VSA increases further (1000–2000 m^2^/cm^3^), *N_DES_* rises sharply, indicating that an increased VSA enhances the MOF adsorption performance. When VSA falls within 2000–2750 m^2^/cm^3^, the growth rate of *N_DES_* slows but does not show a notable decline. At this point, the MOF pore structure is sufficient to accommodate a significant number of DES molecules, so further increases in VSA contribute minimally to adsorption performance. Additionally, the color scale in the figure illustrates variations in *ϕ*. In areas with low *ϕ* (purple and dark blue), *N_DES_* remains low even with a high VSA, suggesting that lower *ϕ* limits the adsorption capacity of MOFs. As *ϕ* increases (transitioning to lighter blue and green), *N_DES_* rises significantly, indicating that higher *ϕ* enables more effective utilization of the VSA in MOFs, enhancing adsorption performance. It is worth noting that although MOFs with a larger VSA tend to exhibit higher *ϕ*, this relationship is not strictly linear. In certain cases, MOFs with a large VSA may not have the highest porosity, which may be influenced by the material’s microstructure and topology.

Selectivity(S) is a key indicator of MOFs’ adsorption–separation performance, reflecting their capacity to preferentially adsorb target molecules. High selectivity suggests a MOF’s tendency to absorb specific molecules, widely applicable in gas separations. Given the difference in magnitude between selectivity and adsorption amount, we use Shah et al.’s [64] TSN metric to evaluate overall adsorption, with details on S-descriptor relationships and TSN calculations in the Supplementary Materials. TSN serves as the color map in Figure 1c and subsequent figures, where higher values indicate better overall adsorption. To ensure analysis validity, MOFs with TSN < 0 were excluded, leaving 13,307 MOFs with TSN > 0 for analysis. Figure 1c shows that as *N_DES_* initially increases, ln(S) rises sharply, indicating that at low adsorption levels, MOFs tend to preferentially adsorb DES molecules with high selectivity. As *N_DES_* increases further, N_2_ and O_2_ also enter the pores, reducing selectivity. This suggests that moderate selectivity combined with high adsorption optimizes TSN. Figure 1d shows that low-density MOFs (552 < *ρ* < 857 kg/m^3^) achieve higher *N_DES_* due to larger pore structures that facilitate DES entry and interaction. As density increases (*ρ* > 593 kg/m^3^), *N_DES_* quickly declines, as higher-density MOFs have smaller pores and reduced contact with DES molecules, decreasing adsorption efficiency. Figure 1e illustrates the relationship between $Q_{st}^{0}$ and *N_DES_*. When $Q_{st}^{0}$ ≤ 25 kJ/mol, adsorption is low; as $Q_{st}^{0}$ rises to 25–50 kJ/mol, *N_DES_* increases, indicating stronger interactions. This trend is consistent with findings from previous studies [65,66,67,68]. Figure 1f shows the relationship between PLD and *N_DES_*, resembling the trend with LCD. As PLD increases, *N_DES_* rises, but excessive PLD causes *N_DES_* to decline, further confirming the crucial role of pore size in adsorption performance.

In studying material adsorption performance, understanding only the correlations between feature descriptors and adsorption capacity can be overly simplistic. To gain deeper insights, MOFs were categorized into three groups: Top materials (TSN > 70 and *N_DES_* > 6 mol/kg), Bottom materials (TSN < 20 and *N_DES_* < 2 mol/kg), and Middle materials (between these thresholds), with structural feature ranges identified for both Top and Bottom materials. This classification is based on observations that materials with TSN values above 70 generally exhibit adsorption capacities over 6 mol/kg, considered a marker of high performance, while materials with TSN values below 20 typically have adsorption capacities under 2 mol/kg [22,69]. Differences in adsorption performance primarily stem from the structural composition of MOFs. Table 1 provides a comparison of structural feature ranges across different MOFs.

As seen in Table 1, Top materials generally have a high *ϕ*, moderate VSA and pore sizes, and relatively low *ρ*, indicating a pore structure that is more conducive to molecular access and adsorption, resulting in excellent adsorption performance. In contrast, Middle materials exhibit a broader distribution range for these characteristics, displaying significant heterogeneity and lower stability in adsorption performance. The structural parameters of Bottom materials are even more varied; while some materials possess a large VSA and *ϕ*, mismatched characteristics like pore size and *ρ* limit their adsorption performance significantly. Additionally, Bottom materials with excessively large or small pore sizes may hinder effective molecular adsorption, while higher *ρ* further reduces adsorption efficiency. Overall, the structural characteristics of Top materials are more balanced, making them well-suited for optimized adsorption performance, whereas the adsorption potential of Middle and Bottom materials is constrained by their diverse and unstable structural features.

By analyzing the structure–performance relationships of all MOF materials and categorizing different performance ranges based on adsorption efficiency, we can gain deeper insights into how descriptor parameters affect adsorption performance. This approach also reveals the performance characteristics of materials under various conditions, providing clear guidance for optimizing material design. However, the limitation of univariate analysis lies in its focus on the independent effects of a few variables, which fails to comprehensively reveal the complex interrelations among multiple variables and their combined impact on TSN. Therefore, this study further employs multivariate analysis with ML techniques to simultaneously examine the interactions among multiple variables and their effects on TSN, providing a more scientifically grounded basis for precise material design and optimization.

### 3.2. Machine Learning

While univariate analysis provides a basic understanding of the structure–performance relationship in DES adsorption by MOF materials, machine learning algorithms allow for a deeper exploration of the relationships between structure and performance, helping to identify key features that can effectively guide structural design for CWA adsorption. This study used 16 feature descriptors as inputs for the models; further details are provided in Table S4. Based on the CoRE-MOFs dataset, four machine learning models were developed: Random Forest (RF), CatBoost, XGBoost (XGB), and LightGBM (LGBM). These algorithms were tested for accuracy in predicting MOFs adsorption performance, and feature importance analysis was further conducted to reveal each feature’s relative contribution to the model’s output.

**Figure 2:**
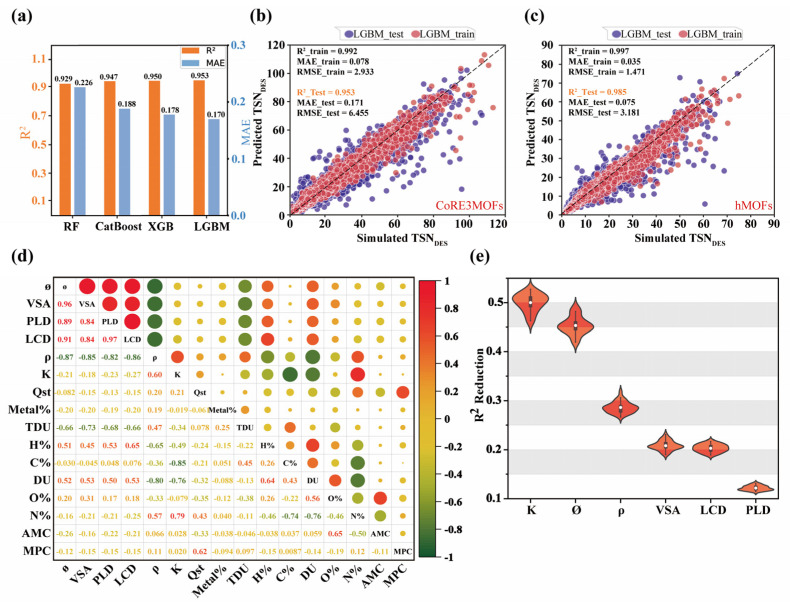
(**a**) R^2^ and MAE comparison of four ML algorithms with TSN as the target on the test set. (**b**) and (**c**): predicted vs. simulated TSN for the CoRE3MOFs and hMOFs datasets, respectively. (**d**) Correlation matrix of adsorption properties and structural parameters. (**e**) The impact of feature permutation on R^2^.

As shown in Figure 2a, the comparison of the R^2^ and MAE results on the test set for these four algorithms reveals that LGBM achieved the highest R^2^ (0.953) and the lowest MAE (0.170), indicating the best performance among the four algorithms. Therefore, the LGBM model was selected as the primary tool for subsequent analysis. Figure 2b illustrates the predictive performance of the LGBM model on the CoRE-MOFs dataset, with an R^2^ of 0.992 and an MAE of 0.078 on the training set, demonstrating an excellent fit to the training data. On the test set, the R^2^ was 0.953, and the MAE was 0.171. Although slightly lower than the training set, the model’s predictive performance remained impressive. Most points in the plot are distributed near the y = x diagonal line, further confirming the model’s high accuracy. Although errors increased in the high TSN value region of the test set, the model’s overall performance remained stable.

To validate the model’s generalization ability, the trained LGBM model was applied to a completely new hMOF dataset. Figure 2c shows the model’s performance on the hMOFs dataset, achieving an R^2^ of 0.997 on the training set and 0.985 on the test set, indicating exceptionally high accuracy with new data. To further explore the model’s transferability to new data, an uncertainty analysis was conducted. Results showed an uncertainty of 1.27 × 10^−15^ in the new dataset, significantly lower than the 2.76 × 10^−15^ observed in the original dataset. This indicates that the model’s predictions on the new dataset are more stable and less variable, likely due to the closer similarity of the hMOF dataset to the training data. Thus, the model demonstrated strong adaptability to the new dataset, further confirming its excellent transferability and high predictive accuracy across different datasets.

Figure 2d presents a Pearson correlation matrix, revealing relationships among MOF features and performance metrics. VSA and *ϕ* have a correlation coefficient of 0.96, indicating that the VSA significantly increases with *ϕ*, thereby enhancing surface contact with adsorbate molecules, consistent with the results from univariate analysis. The negative correlation between LCD and *ρ* (−0.86) shows that density decreases as pore size increases, reflecting the structural porosity of MOFs. Meanwhile, the correlation of 0.60 between K and *ρ* suggests that denser materials typically exhibit a higher Henry’s coefficient, enhancing adsorption through stronger interactions at adsorption sites. Many studies simplify models by removing highly correlated features identified via the Pearson correlation matrix. However, our results show a decline in the LGBM model’s R^2^ value after removing features with correlations above 0.85 or below −0.85, suggesting that these features may uniquely contribute to model accuracy despite their high correlations. To assess the actual importance of these highly correlated features, Permutation Feature Importance (PFI) was applied to six key features: K, *ϕ*, *ρ*, VSA, PLD, and LCD (see Supplementary Materials for details). Figure 2e shows violin plots depicting the impact of 30 random permutations for each feature on the model’s R^2^ value, revealing substantial variability in feature importance. Overall, Figure 2e highlights the relative importance of each feature in maintaining the model’s accuracy. Despite the high correlations among certain features, their unique contributions warrant retention in the model, consistent with previous findings [70].

**Figure 3:**
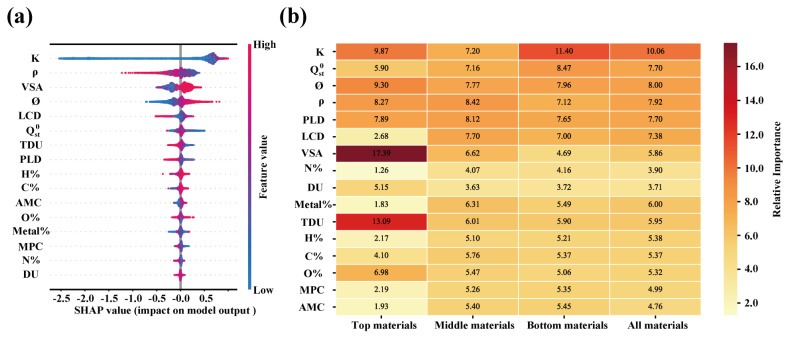
(**a**) SHAP analysis showing feature impact on model output. (**b**) Relative feature importance comparison for Top, Middle, and Low materials.

To further evaluate the importance of each feature in performance prediction, Shapley Additive Explanations (SHAP) values were utilized for interpretive analysis, as shown in Figure 3a. The results indicate that K, *ρ*, VSA, and *ϕ* are the most influential features on the model output, with K exhibiting the widest SHAP value range, signifying its largest contribution to the prediction results [71]. High SHAP values (in red) indicate a significant positive impact of K on TSN. The SHAP distribution for *ρ*, however, shows a more complex effect, with both positive and negative influences, suggesting that while density within a certain range can enhance adsorption, overly high density may have a limiting effect. In comparison, the SHAP values for VSA and *ϕ* suggest that larger values for these features increase the contact area for adsorbate molecules, significantly enhancing adsorption performance. Additionally, features such as Metal% and N% show lower SHAP values, indicating a limited impact on the model. Higher concentrations of H%, C%, and O% are distributed to the right of zero, indicating a positive contribution of these elements to the adsorption performance. This suggests that organic ligands containing moderate amounts of H, C, and O are beneficial for improving adsorption performance. This analysis effectively quantifies the impact of each feature on the model, revealing the roles of various features in predicting TSN. In further analysis, feature importance was calculated after categorizing the material database, as shown in Figure 3b. VSA and TDU play a critical role in the adsorption performance of Top materials, with VSA’s importance far exceeding that of other features, indicating that a larger VSA significantly enhances adsorption capacity. TDU also has a notable impact on Top materials, suggesting that structures with high unsaturation are crucial for adsorption performance [72]. However, the influence of these two features is significantly reduced in Middle and Bottom materials. Furthermore, K has the highest relative importance in Bottom materials, reaching 11.40, indicating that in lower-performance materials, the K feature contributes more significantly to the adsorption capacity. Additionally, *ϕ* shows an importance of 9.30 in Top materials, significantly higher than in the other two categories, indicating that optimal porosity is essential for enhancing the adsorption capacity of Top materials. Overall, VSA and TDU are the most important features in Top materials, while the significance of features such as K and *ϕ* increases in Middle and Bottom materials. Furthermore, the importance ranking of the four descriptor categories across the three material classes is as follows: structural descriptors > chemical descriptors > energy descriptors > charge descriptors. Therefore, in designing higher-performance materials, it is essential to prioritize achieving a high VSA and adequate total unsaturation.

### 3.3. Molecular Fingerprint

Although the above analytical methods can identify promising MOF adsorbents from a large dataset and highlight the importance of feature descriptors for DES adsorption, the specific influence of MOFs’ functional groups and molecular structures on adsorption performance remains an underexplored issue at the molecular design level. For instance, carbon can exist in aromatic or aliphatic forms, while nitrogen can appear in cyano ligands or imidazole rings. This structural diversity often has a significant impact on the adsorption and separation behavior of target molecules [69].

To quantify the influence of MOFs’ functional groups and molecular structures on adsorption performance at the material design level, we applied a molecular fingerprinting algorithm to transform all the material samples. We then analyzed the occurrence frequencies of the best- and worst-performing fingerprints across Top, Middle, and Bottom materials to uncover the distribution patterns of fingerprint features among different performance categories. For all three categories, fingerprint bits with occurrence frequencies exceeding 80% (e.g., 165, 160, 147, 141, and 137) were defined as ordinary bits. Since these bits are ubiquitous across materials, they do not effectively distinguish the unique characteristics of MOFs and were thus excluded from the analysis to reduce noise and ensure accuracy. Details on the noise threshold definition and calculation process for each material group are provided in the Supporting Information (SI). Based on a 50% threshold, unique fingerprints for Top materials were identified, including bits 164, 146, 163, 126, 158, and 124 (see Table S4 for details). These fingerprints appeared in more than 50% of Top materials but in less than 30% of Bottom materials, suggesting their positive contribution to adsorption performance. Conversely, fingerprints with occurrence frequencies below 50% in Top materials but exceeding 70% in Bottom materials were inferred to negatively affect adsorption performance. However, no such negatively correlated fingerprints were found in this analysis. This frequency-based selection method is widely used in data mining and feature selection to reduce noise and enhance both the stability and predictive performance of models [73,74,75].

Furthermore, this study builds on previous statistical analysis methods for molecular fingerprints to complement the findings [45]. Given the significant sample size differences between the two material groups, the bit difference method was introduced to calculate relative errors, enabling more effective identification of frequently occurring fingerprint features in each category. The results show that fingerprint bits 131, 125, 66, 122, 138, 79, 121, 120, and 162 have significantly higher relative frequencies in Bottom materials compared to Top and Overall materials, with differences ranging from 15% to 20%. Therefore, these bits are defined as fingerprints that negatively impact performance. Ultimately, by combining these two analytical methods, we comprehensively identified molecular fingerprints associated with material performance, providing robust guidance for more precise material design.

**Figure 4:**
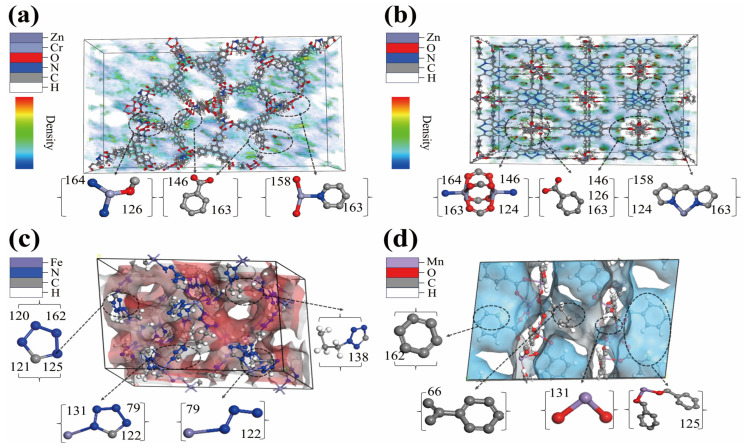
(**a**) and (**b**) show the adsorption density maps of Top-material MOFs, SETSUH and ILITON, respectively. (**c**) WILNEL and (**d**) VUQJUN represent Bottom-material MOFs.

Based on the aforementioned fingerprint frequency analysis, two MOFs, SETSUH and ILITON, which contain all high-performance fingerprints, were selected from the Top materials. Their adsorption density distributions were visualized for further analysis and interpretation. Figure 4a,b illustrate the key fingerprint features of these MOFs. The results reveal that DES molecules exhibit higher density distributions at the connection points of C–N single-bonded six-membered rings and metal oxides, which are closely associated with the presence of open metal sites. Open metal sites, due to incomplete coordination, exhibit high electron affinity and reactivity, making them prone to forming adsorption or weak coordination interactions with nearby molecules (e.g., DES). This interaction induces local polarization effects, leading to concentrated electron cloud distributions near the C–N single-bonded six-membered rings, thereby exhibiting higher density characteristics [70,76,77,78]. Studies have shown that introducing open metal sites in MOF pores under low-concentration gas conditions significantly enhances interactions with CWAs and is widely regarded as an effective gas capture strategy [15]. Furthermore, the synergistic effect between open metal sites and functional groups enhances the adsorption performance of materials by providing additional strong adsorption sites for target molecules, thereby significantly improving capture efficiency [79]. The figures show that most DES molecules aggregate around polycyclic structures, nitrogen-containing heterocycles, C=C double bonds, active metal sites, and oxygen atoms. This indicates that MOF organic ligands featuring these high-quality fingerprints induce significant changes in the electronic environment, enhancing the affinity for DES molecules and significantly improving adsorption performance [67,78,80]. Additionally, some DES molecules tend to aggregate at the center of the pores, likely due to the synergistic effects of multiple functional groups on the pore walls. These functional groups generate significant synergistic potential energy at the pore center, thereby effectively attracting DES molecules. Overall, the density of DES molecules is highest around functional groups, while moderate density is observed at the pore center, further demonstrating the critical role of functional groups in determining MOF adsorption performance.

Two low-performance MOFs, WILNEL and VUQJUN, were selected from the Bottom materials. Figure 4c,d provide a structural breakdown of these low-performance MOFs. The analysis reveals that MOFs containing only a single fingerprint feature, such as six-membered rings, aromatic rings, or nitrogen-containing heterocycles, exhibit suboptimal adsorption performance. Furthermore, metal cluster structures connected to nitrogen generally exhibit lower adsorption performance than those connected to oxygen, as the presence of oxygen atoms tends to enhance the affinity of the metal clusters [81]. Previous studies on MOF functional groups have largely relied on experimental approaches, often using trial-and-error methods to identify critical functional groups, such as OH–, NH_2_, CHO–, pyridine, imidazole, pyrazine, and triazole. However, this process is limited by small sample sizes and low efficiency [82,83,84,85,86]. Currently, fingerprinting techniques enable more efficient identification of key functional groups, significantly accelerating material design and screening processes while enhancing the accuracy and efficiency of the analysis.

## 4. Conclusions

MOFs have shown significant potential for removing CWAs from the air. This study combines molecular simulations, machine learning, and molecular fingerprinting techniques to identify high-performance MOFs for DES adsorption and investigate the relationship between their structure and performance. First, univariate analysis was conducted to explore the relationship between six key descriptors (LCD, VSA, PLD, and K) and MOFs adsorption performance. MOFs were classified into Top materials (TSN > 70; *N_DES_* > 6 mol/kg), Bottom materials (TSN < 20; *N_DES_* < 2 mol/kg), and Middle materials (falling in between), thereby delineating the structural characteristic ranges for each performance category. Next, four machine learning methods were employed to predict and analyze MOFs adsorption performance. The results revealed that TSN is a more suitable performance metric compared to *N_DES_* and S, with the LGBM model achieving the best predictive performance. Feature contribution analysis using the SHAP algorithm identified K, *ρ*, VSA, and *ϕ* as the most critical features influencing the model’s predictions. Moreover, the importance of features varied across performance categories: VSA and TDU contributed most significantly to Top materials, while K and *ϕ* were more prominent in Middle and Bottom materials. This analysis revealed the key factors and feature differences influencing adsorption performance for each category. Overall, the importance of the four descriptor categories was ranked as follows: structural descriptors > chemical descriptors > energy descriptors > charge descriptors. Finally, molecular fingerprint statistical methods and adsorption density analysis were employed to identify and compare fingerprint features between Top and Bottom materials. The results showed that MOFs containing multiple key fingerprints, such as metal oxides, nitrogen-containing heterocycles, six-membered rings, and C=C double bonds, exhibited significantly improved adsorption performance. This study provides not only theoretical guidance for the synthesis and optimization of MOFs but also valuable insights into the design of efficient CWA adsorption materials.

## Figures and Tables

**Table 1:** Range of structural and energetic descriptors for Top, Middle, and Bottom materials.

	Top Materials	Middle Materials	Bottom Materials
*ϕ*	0.164–0.975	0.118–0.998	0.041–0.988
VSA	1555.04–3141.57	0–3213.68	0–3557.18
PLD	4.219–14.932	0–28.611	0–38.066
LCD	5.632–23.603	0–30.095	0–39.111
*ρ*	516.245–1045.01	353.615–2199.76	56.920–3499.51
K	0.003–2.98 × 10^9^	0.001–4.97 × 10^16^	3.97 × 10^−12^–1.47 × 10^44^
$Q_{st}^{0}$	30.723–107.024	26.324–164.508	−2.852–190.494

## Data Availability

The data supporting the findings of this study are not publicly available due to institutional restrictions. However, they may be available from the corresponding author upon reasonable request and with appropriate permissions.

## Supplementary Materials

The following supporting information can be downloaded at https://www.mdpi.com/article/10.3390/nano15030183/s1: Table S1. Lennard–Jones parameters of MOF; Table S2. Lennard–Jones parameters and charges of adsorbates; Table S3. Critical constants for CWAs and simulants; Table S4. Detailed information of descriptors for the four types; Table S5. Hyperparameters obtained by grid search with cross-validation; Table S6. Evaluation metrics for four ML algorithms; Table S7 Evaluation of each ML algorithm by running the algorithm five times; Table S8. Detailed information about the bits; Figure S1. Schematic diagram of one-hot encoding; Figure S2. Descriptors–performance relationship between (a) LCD, lnS, and TSN, (b) *ϕ*, lnS, and TSN, (c) PLD, lnS, and TSN, (d) *Q*0st, lnS, and TSN, (e) VSA, lnS, and TSN, and (f) *ρ*, lnS, and TSN; Figure S3. Random Forest regression; Figure S4. Extreme Gradient Boosting regression; Figure S5. Light Gradient Boosting Machine regression; Figure S6. CatBoost algorithm flowchart; Figure S7. The schematic diagram of Bayesian optimization; Figure S8. The diagram of k-fold cross-validation; Figure S9. Feature importance based on SHAP values; Figure S10. Clausius−Clapeyron plot for DMMP (circle), DMMP-VN (diamond), sarin (square), and soman (triangle); Figure S11. Absolute value of the enthalpy of adsorption at infinite dilution ΔH of sarin and simulants in MOFs at 298 K; Figure S12. Surface tension of DMMP; Figure S13. Discussion of simulation reliability for MOFs with open metal sites under generic force fields; Figure S14. Comparison of the experimental CH4 isotherms with isotherms calculated; Figure S15. Comparison of simulated adsorption isotherms in Mg-MOF-74 with the experimental one; Figure S16. Adsorption isotherms of CO_2_ and N2 at 298 K; Figure S17. CO_2_ isotherms in various MOFs. References [87,88,89,90,91,92,93,94,95,96] are cited in the Supplementary Materials.

## References

[1] Wattana M., Bey T. (2009). Mustard Gas or Sulfur Mustard: An Old Chemical Agent as a New Terrorist Threat. Prehosp. Disaster Med..

[2] Couzon N., Dhainaut J., Campagne C., Royer S., Loiseau T., Volkringer C. (2022). Porous Textile Composites (PTCs) for the Removal and the Decomposition of Chemical Warfare Agents (CWAs)—A Review. Coord. Chem. Rev..

[3] Liu Y., Howarth A.J., Vermeulen N.A., Moon S.-Y., Hupp J.T., Farha O.K. (2017). Catalytic Degradation of Chemical Warfare Agents and Their Simulants by Metal-Organic Frameworks. Coord. Chem. Rev..

[4] Sambrook M.R., Notman S. (2013). Supramolecular Chemistry and Chemical Warfare Agents: From Fundamentals of Recognition to Catalysis and Sensing. Chem. Soc. Rev..

[5] Prasad G.K., Singh B., Saradhi U.V.R., Suryanarayana M.V.S., Pandey D. (2002). Adsorption and Reaction of Diethyl Sulfide on Active Carbons with and without Impregnants under Static Conditions. Langmuir.

[6] Snider V.G., Hill C.L. (2023). Functionalized Reactive Polymers for the Removal of Chemical Warfare Agents: A Review. J. Hazard. Mater..

[7] Grissom T.G., Sirrine J.M., Long T.E., Esker A.R., Morris J.R. (2017). Interaction Parameters for the Uptake of Sulfur Mustard Mimics into Polyurethane Films. Prog. Org. Coat..

[8] De Gisi S., Lofrano G., Grassi M., Notarnicola M. (2016). Characteristics and Adsorption Capacities of Low-Cost Sorbents for Wastewater Treatment: A Review. Sustain. Mater. Technol..

[9] Demirbas E., Nas M.Z. (2009). Batch Kinetic and Equilibrium Studies of Adsorption of Reactive Blue 21 by Fly Ash and Sepiolite. Desalination.

[10] Kim K., Tsay O.G., Atwood D.A., Churchill D.G. (2011). Destruction and Detection of Chemical Warfare Agents. Chem. Rev..

[11] Furukawa H., Ko N., Go Y.B., Aratani N., Choi S.B., Choi E., Yazaydin A.Ö., Snurr R.Q., O’Keeffe M., Kim J. (2010). Ultrahigh Porosity in Metal-Organic Frameworks. Science.

[12] Fan W., Zhang X., Kang Z., Liu X., Sun D. (2021). Isoreticular Chemistry within Metal–Organic Frameworks for Gas Storage and Separation. Coord. Chem. Rev..

[13] Trickett C.A., Helal A., Al-Maythalony B.A., Yamani Z.H., Cordova K.E., Yaghi O.M. (2017). The Chemistry of Metal–Organic Frameworks for CO_2_ Capture, Regeneration and Conversion. Nat. Rev. Mater..

[14] Mondloch J.E., Katz M.J., Iii W.C.I., Ghosh P., Liao P., Bury W., Wagner G.W., Hall M.G., DeCoste J.B., Peterson G.W. (2015). Destruction of Chemical Warfare Agents Using Metal–Organic Frameworks. Nat. Mater..

[15] Islamoglu T., Chen Z., Wasson M.C., Buru C.T., Kirlikovali K.O., Afrin U., Mian M.R., Farha O.K. (2020). Metal–Organic Frameworks against Toxic Chemicals. Chem. Rev..

[16] Ullah S., McKee M.L., Samokhvalov A. (2023). A Zinc-Containing Porphyrin Aluminum MOF in Sorption of Diethyl Sulfide Vapor: Mechanistic Experimental and Computational Study. Phys. Chem. Chem. Phys..

[17] Ahmad M., Samokhvalov A. (2024). Porphyrin-Based Aluminum Metal-Organic Framework with Copper: Pre-Adsorption of Water Vapor, Dynamic and Static Sorption of Diethyl Sulfide Vapor, and Sorbent Regeneration. Materials.

[18] Colón Y.J., Snurr R.Q. (2014). High-Throughput Computational Screening of Metal–Organic Frameworks. Chem. Soc. Rev..

[19] Daglar H., Keskin S. (2020). Recent Advances, Opportunities, and Challenges in High-Throughput Computational Screening of MOFs for Gas Separations. Coord. Chem. Rev..

[20] Chong S., Lee S., Kim B., Kim J. (2020). Applications of Machine Learning in Metal-Organic Frameworks. Coord. Chem. Rev..

[21] Qiao Z., Peng C., Zhou J., Jiang J. (2016). High-Throughput Computational Screening of 137953 Metal–Organic Frameworks for Membrane Separation of a CO_2_/N_2_/CH_4_ Mixture. J. Mater. Chem..

[22] Qiao Z., Xu Q., Jiang J. (2018). Computational Screening of Hydrophobic Metal–Organic Frameworks for the Separation of H_2_S and CO_2_ from Natural Gas. J. Mater. Chem. A.

[23] Qiao Z., Xu Q., Jiang J. (2018). High-Throughput Computational Screening of Metal-Organic Framework Membranes for Upgrading of Natural Gas. J. Membr. Sci..

[24] Agrawal M., Sava Gallis D.F., Greathouse J.A., Sholl D.S. (2018). How Useful Are Common Simulants of Chemical Warfare Agents at Predicting Adsorption Behavior?. J. Phys. Chem. C.

[25] Wang G., Sharp C., Plonka A.M., Wang Q., Frenkel A.I., Guo W., Hill C., Smith C., Kollar J., Troya D. (2017). Mechanism and Kinetics for Reaction of the Chemical Warfare Agent Simulant, DMMP(*g*), with Zirconium(IV) MOFs: An Ultrahigh-Vacuum and DFT Study. J. Phys. Chem. C.

[26] Soares C.V., Leitão A.A., Maurin G. (2019). Computational Evaluation of the Chemical Warfare Agents Capture Performances of Robust MOFs. Micropor. Mesopor. Mater..

[27] Ullah S., McKee M.L., Samokhvalov A. (2023). Interaction of a Porphyrin Aluminum Metal–Organic Framework with Volatile Organic Sulfur Compound Diethyl Sulfide Studied via in Situ and Ex Situ Experiments and DFT Computations. Nanomaterials.

[28] Yan Y., Shi Z., Li H., Li L., Yang X., Li S., Liang H., Qiao Z. (2022). Machine Learning and In-Silico Screening of Metal–Organic Frameworks for O_2_/N_2_ Dynamic Adsorption and Separation. Chem. Eng. J..

[29] Shi Z., Yang W., Deng X., Cai C., Yan Y., Liang H., Liu Z., Qiao Z. (2020). Machine-Learning-Assisted High-Throughput Computational Screening of High Performance Metal–Organic Frameworks. Mol. Syst. Des. Eng..

[30] Demir H., Daglar H., Gulbalkan H.C., Aksu G.O., Keskin S. (2023). Recent Advances in Computational Modeling of MOFs: From Molecular Simulations to Machine Learning. Coord. Chem. Rev..

[31] Jablonka K.M., Ongari D., Moosavi S.M., Smit B. (2020). Big-Data Science in Porous Materials: Materials Genomics and Machine Learning. Chem. Rev..

[32] Mortazavi B. (2024). Recent Advances in Machine Learning-assisted Multiscale Design of Energy Materials. Adv. Energy Mater..

[33] Demir H., Keskin S. (2024). A New Era of Modeling MOF-based Membranes: Cooperation of Theory and Data Science. Macromol. Mater. Eng..

[34] Daglar H., Gulbalkan H.C., Aksu G.O., Keskin S. (2024). Computational Simulations of Metal–Organic Frameworks to Enhance Adsorption Applications. Adv. Mater..

[35] Neikha K., Puzari A. (2024). Metal–Organic Frameworks through the Lens of Artificial Intelligence: A Comprehensive Review. Langmuir.

[36] Lin J., Liu Z., Guo Y., Wang S., Tao Z., Xue X., Li R., Feng S., Wang L., Liu J. (2023). Machine Learning Accelerates the Investigation of Targeted MOFs: Performance Prediction, Rational Design and Intelligent Synthesis. Nano Today.

[37] Coudert F.-X., Fuchs A.H. (2016). Computational Characterization and Prediction of Metal–Organic Framework Properties. Coord. Chem. Rev..

[38] Mai H., Le T.C., Chen D., Winkler D.A., Caruso R.A. (2022). Machine Learning in the Development of Adsorbents for Clean Energy Application and Greenhouse Gas Capture. Adv. Sci..

[39] Zhang X., Xu Z., Wang Z., Liu H., Zhao Y., Jiang S. (2023). High-Throughput and Machine Learning Approaches for the Discovery of Metal Organic Frameworks. APL Mater..

[40] Bucior B.J., Bobbitt N.S., Islamoglu T., Goswami S., Gopalan A., Yildirim T., Farha O.K., Bagheri N., Snurr R.Q. (2019). Energy-Based Descriptors to Rapidly Predict Hydrogen Storage in Metal–Organic Frameworks. Mol. Syst. Des. Eng..

[41] Yang X., Huang Q., Zhang L., Li L., Chen Y., Wang W., Liang H., Wu Y., Zheng H., Zhao Y. (2023). Computational Screening and Machine Learning of Hydrophobic Metal-Organic Frameworks for Removal of Chemical Warfare Agents from Air. Appl. Mater. Today.

[42] Wang W., Zhang L., Cai C., Li S., Liang H., Wu Y., Zheng H., Qiao Z. (2023). Machine Learning Assisted High-Throughput Computational Screening of MOFs for the Capture of Chemical Warfare Agents from the Air. Sep. Purif. Technol..

[43] Lin Z., Cai H., Peng H., Fang Y., Pan P., Li H., Yang Y., Yao J. (2024). Enhancing Arsenate Removal through Interpretable Machine Learning Guiding the Modular Design of Metal–Organic Frameworks. Chem. Eng. J..

[44] Yuan X., Li L., Shi Z., Liang H., Li S., Qiao Z. (2022). Molecular-Fingerprint Machine-Learning-Assisted Design and Prediction for High-Performance MOFs for Capture of NMHCs from Air. Adv. Powder Mater..

[45] Ni J., Li J., Li S., Zheng H., Ming Z., Li L., Li H., Zhang S., Zhao Y., Liang H. (2024). Molecular Fingerprint and Machine Learning Enhance High-Performance MOFs for Mustard Gas Removal. iScience.

[46] Matito-Martos I., Moghadam P.Z., Colombo V., Navarro J.A.R., Calero S., Fairen-Jimenez D. (2018). Discovery of an Optimal Porous Crystalline Material for the Capture of Chemical Warfare Agents. Chem. Mater..

[47] Chung Y.G., Haldoupis E., Bucior B.J., Haranczyk M., Lee S., Zhang H., Vogiatzis K.D., Milisavljevic M., Ling S., Camp J.S. (2019). Advances, Updates, and Analytics for the Computation-Ready, Experimental Metal–Organic Framework Database: CoRE MOF 2019. J. Chem. Eng. Data.

[48] Bobbitt N.S., Shi K., Bucior B.J., Chen H., Tracy-Amoroso N., Li Z., Sun Y., Merlin J.H., Siepmann J.I., Siderius D.W. (2023). MOFX-DB: An Online Database of Computational Adsorption Data for Nanoporous Materials. J. Chem. Eng. Data.

[49] Wilmer C.E., Leaf M., Lee C.Y., Farha O.K., Hauser B.G., Hupp J.T., Snurr R.Q. (2011). Large-Scale Screening of Hypothetical Metal-Organic Frameworks. Nat. Chem..

[50] Willems T.F., Rycroft C.H., Kazi M., Meza J.C., Haranczyk M. (2012). Algorithms and Tools for High-Throughput Geometry-Based Analysis of Crystalline Porous Materials. Microporous Mesoporous Mater..

[51] Dubbeldam D., Calero S., Ellis D.E., Snurr R.Q. (2016). RASPA: Molecular Simulation Software for Adsorption and Diffusion in Flexible Nanoporous Materials. Mol. Simulat..

[52] Sokkalingam N., Kamath G., Coscione M., Potoff J.J. (2009). Extension of the Transferable Potentials for Phase Equilibria Force Field to Dimethylmethyl Phosphonate, Sarin, and Soman. J. Phys. Chem. B.

[53] Wilmer C.E., Kim K.C., Snurr R.Q. (2012). An Extended Charge Equilibration Method. J. Phys. Chem. Lett..

[54] Liu A., Peng X., Jin Q., Jain S.K., Vicent-Luna J.M., Calero S., Zhao D. (2019). Adsorption and Diffusion of Benzene in Mg-MOF-74 with Open Metal Sites. ACS Appl. Mater. Interfaces.

[55] Koh H.S., Rana M.K., Wong-Foy A.G., Siegel D.J. (2015). Predicting Methane Storage in Open-Metal-Site Metal–Organic Frameworks. J. Phys. Chem. C.

[56] Rappe A.K., Casewit C.J., Colwell K.S., Goddard W.A., Skiff W.M. (1992). UFF, a Full Periodic Table Force Field for Molecular Mechanics and Molecular Dynamics Simulations. J. Am. Chem. Soc..

[57] Lundberg S.M., Lee S.-I. (2017). A Unified Approach to Interpreting Model Predictions. arXiv.

[58] O’Boyle N.M., Banck M., James C.A., Morley C., Vandermeersch T., Hutchison G.R. (2011). Open Babel: An Open Chemical Toolbox. J. Cheminf..

[59] Yap C.W. (2011). PaDEL-Descriptor: An Open Source Software to Calculate Molecular Descriptors and Fingerprints. J. Comput. Chem..

[60] Durant J.L., Leland B.A., Henry D.R., Nourse J.G. (2002). Reoptimization of MDL Keys for Use in Drug Discovery. J. Chem. Inf. Comput. Sci..

[61] Bucior B.J., Rosen A.S., Haranczyk M., Yao Z., Ziebel M.E., Farha O.K., Hupp J.T., Siepmann J.I., Aspuru-Guzik A., Snurr R.Q. (2019). Identification Schemes for Metal–Organic Frameworks To Enable Rapid Search and Cheminformatics Analysis. Cryst. Growth Des..

[62] Watanabe T., Sholl D.S. (2012). Accelerating Applications of Metal–Organic Frameworks for Gas Adsorption and Separation by Computational Screening of Materials. Langmuir.

[63] Furukawa H., Cordova K.E., O’Keeffe M., Yaghi O.M. (2013). The Chemistry and Applications of Metal-Organic Frameworks. Science.

[64] Shah M.S., Tsapatsis M., Siepmann J.I. (2016). Identifying Optimal Zeolitic Sorbents for Sweetening of Highly Sour Natural Gas. Angew. Chem. Int. Ed..

[65] Liang H., Yang W., Peng F., Liu Z., Liu J., Qiao Z. (2019). Combining Large-Scale Screening and Machine Learning to Predict the Metal-Organic Frameworks for Organosulfurs Removal from High-Sour Natural Gas. APL Mater..

[66] Qiao Z., Yan Y., Tang Y., Liang H., Jiang J. (2021). Metal–Organic Frameworks for Xylene Separation: From Computational Screening to Machine Learning. J. Phys. Chem. C.

[67] Yuan X., Deng X., Cai C., Shi Z., Liang H., Li S., Qiao Z. (2021). Machine Learning and High-Throughput Computational Screening of Hydrophobic Metal–Organic Frameworks for Capture of Formaldehyde from Air. Green Energy Environ..

[68] Bai X., Shi Z., Xia H., Li S., Liu Z., Liang H., Liu Z., Wang B., Qiao Z. (2022). Machine-Learning-Assisted High-Throughput Computational Screening of Metal–Organic Framework Membranes for Hydrogen Separation. Chem. Eng. J..

[69] Gao W., Zheng W., Yan K., Sun W., Zhao L. (2023). Accelerating the Discovery of Acid Gas-Selective MOFs for Natural Gas Purification: A Combination of Machine Learning and Molecular Fingerprint. Fuel.

[70] Guan K., Xu F., Huang X., Li Y., Guo S., Situ Y., Chen Y., Hu J., Liu Z., Liang H. (2024). Deep Learning and Big Data Mining for Metal–Organic Frameworks with High Performance for Simultaneous Desulfurization and Carbon Capture. J. Colloid Interface Sci..

[71] Guo S., Huang X., Situ Y., Huang Q., Guan K., Huang J., Wang W., Bai X., Liu Z., Wu Y. (2023). Interpretable Machine-Learning and Big Data Mining to Predict Gas Diffusivity in Metal-Organic Frameworks. Adv. Sci..

[72] Kökçam-Demir Ü., Goldman A., Esrafili L., Gharib M., Morsali A., Weingart O., Janiak C. (2020). Coordinatively Unsaturated Metal Sites (Open Metal Sites) in Metal–Organic Frameworks: Design and Applications. Chem. Soc. Rev..

[73] Zhang T., Zhu T., Xiong P., Huo H., Tari Z., Zhou W. (2020). Correlated Differential Privacy: Feature Selection in Machine Learning. IEEE Trans. Ind. Inf..

[74] Li J., Cheng K., Wang S., Morstatter F., Trevino R.P., Tang J., Liu H. (2018). Feature Selection: A Data Perspective. ACM Comput. Surv..

[75] Wu J.M.-T., Zhan J., Chobe S. (2018). Mining Association Rules for Low-Frequency Itemsets. PLoS ONE.

[76] Lee S., Oh S., Lee G., Oh M. (2023). Defective MOF-74 with Ancillary Open Metal Sites for the Enhanced Adsorption of Chemical Warfare Agent Simulants. Dalton Trans..

[77] Li J., Li Y., Situ Y., Wu Y., Wang W., Huang L., Cai C., Huang X., Guan Y., Zhang S. (2024). Unraveling the Separation Mechanism of Gas Mixtures in MOFs by Combining the Breakthrough Curve with Machine Learning and High-Throughput Calculation. Chem. Eng. Sci..

[78] Li Y., Situ Y., Guan K., Guan Y., Huang X., Cai C., Li S., Liu Z., Liang H., Wu Y. (2024). High Dynamic Separation Performance of Metal–Organic Frameworks for D_2_/H_2_: Independent or Competitive Adsorption?. AlChE J..

[79] Britt D., Tranchemontagne D., Yaghi O.M. (2008). Metal-Organic Frameworks with High Capacity and Selectivity for Harmful Gases. Proc. Natl. Acad. Sci. USA.

[80] Huang Q., Yuan X., Li L., Yan Y., Yang X., Wang W., Chen Y., Liang H., Gao H., Wu Y. (2023). Machine Learning and Molecular Fingerprint Screening of High-Performance 2D/3D MOF Membranes for Kr/Xe Separation. Chem. Eng. Sci..

[81] Emelianova A., Reed A., Basharova E.A., Kolesnikov A.L., Gor G.Y. (2023). Closer Look at Adsorption of Sarin and Simulants on Metal–Organic Frameworks. ACS Appl. Mater. Interfaces.

[82] Kraftschik B., Koros W.J., Johnson J.R., Karvan O. (2013). Dense Film Polyimide Membranes for Aggressive Sour Gas Feed Separations. J. Membr. Sci..

[83] Li B., Zhang Z., Li Y., Yao K., Zhu Y., Deng Z., Yang F., Zhou X., Li G., Wu H. (2012). Enhanced Binding Affinity, Remarkable Selectivity, and High Capacity of CO_2_ by Dual Functionalization of a *Rht*-type Metal–Organic Framework. Angew. Chem. Int. Ed..

[84] Couck S., Denayer J.F.M., Baron G.V., Rémy T., Gascon J., Kapteijn F. (2009). An Amine-Functionalized MIL-53 Metal−organic Framework with Large Separation Power for CO_2_ and CH_4_. J. Am. Chem. Soc..

[85] Flaig R.W., Osborn Popp T.M., Fracaroli A.M., Kapustin E.A., Kalmutzki M.J., Altamimi R.M., Fathieh F., Reimer J.A., Yaghi O.M. (2017). The Chemistry of CO_2_ Capture in an Amine-Functionalized Metal–Organic Framework under Dry and Humid Conditions. J. Am. Chem. Soc..

[86] Morris W., Leung B., Furukawa H., Yaghi O.K., He N., Hayashi H., Houndonougbo Y., Asta M., Laird B.B., Yaghi O.M. (2010). A Combined Experimental−computational Investigation of Carbon Dioxide Capture in a Series of Isoreticular Zeolitic Imidazolate Frameworks. J. Am. Chem. Soc..

[87] Lam Pham T., Kino H., Terakura K., Miyake T., Tsuda K., Takigawa I., Chi Dam H. (2017). Machine Learning Reveals Orbital Interaction in Materials. Sci. Technol. Adv. Mater..

[88] Nguyen V. (2019). Bayesian Optimization for Accelerating Hyper-Parameter Tuning. Proceedings of the 2019 IEEE Second International Conference on Artificial Intelligence and Knowledge Engineering (AIKE).

[89] Lundberg S.M., Erion G., Chen H., DeGrave A., Prutkin J.M., Nair B., Katz R., Himmelfarb J., Bansal N., Lee S.-I. (2020). From Local Explanations to Global Understanding with Explainable AI for Trees. Nat Mach Intell.

[90] Fujimoto K., Kojadinovic I., Marichal J.-L. (2006). Axiomatic Characterizations of Probabilistic and Cardinal-Probabilistic Interaction Indices. Games Econ. Behav..

[91] Louw K.I., Bradshaw-Hajek B.H., Hill J.M. (2022). Interaction of Ferric Ions with Europium Metal Organic Framework and Application to Mineral Processing Sensing. Philos. Mag..

[92] Hermann J., DiStasio R.A., Tkatchenko A. (2017). First-Principles Models for van Der Waals Interactions in Molecules and Materials: Concepts, Theory, and Applications. Chem. Rev..

[93] Ivanova E.V., Vasudevan A., Senyurt E.I., Schoenitz M., Khalizov A.F., Dreizin E.L., Gor G.Y. (2023). Surface Tension of Organophosphorus Compounds: Sarin and Its Surrogates. Langmuir.

[94] Krishna R., Van Baten J.M. (2011). In Silico Screening of Metal–Organic Frameworks in Separation Applications. Phys. Chem. Chem. Phys..

[95] Zhang K., Nalaparaju A., Jiang J. (2015). CO_2_ Capture in **Rht** Metal–Organic Frameworks: Multiscale Modeling from Molecular Simulation to Breakthrough Prediction. J. Mater. Chem. A.

[96] Yazaydın A.Ö., Snurr R.Q., Park T.-H., Koh K., Liu J., LeVan M.D., Benin A.I., Jakubczak P., Lanuza M., Galloway D.B. (2009). Screening of Metal−organic Frameworks for Carbon Dioxide Capture from Flue Gas Using a Combined Experimental and Modeling Approach. J. Am. Chem. Soc..

