# Supplementary material for: A Multi-Method Approach to Analyzing MOFs for Chemical Warfare Simulant Capture: Molecular Simulation, Machine Learning, and Molecular Fingerprints
Source: Nanomaterials (Basel). 2025 Jan 24;15(3):183. doi: 10.3390/nano15030183 (PMC11820582; doi:10.3390/nano15030183)
Supplement: Supplementary file 1 [file nanomaterials-15-00183-s001.zip › nanomaterials-3397233-supplementary.pdf]

Supporting Information

A Multi-Method Approach to Analyzing MOFs for Chemical Warfare Simulant Capture: Molecular Simulation, Machine Learning, and Molecular Fingerprints

Zhongyuan Ming [1,2], Min Zhang [1], Shouxin Zhang [1], Xiaopeng Li [1], Xiaoshan Yan [1], Kexin Guan [2], Yu Li [2], Yufeng Peng [2], Jinfeng Li [2], Heguo Li [1,*], Yue Zhao [1,*] and Zhiwei Qiao [2,*]

[1] State Key Laboratory of NBC Protection for Civilian, Institute of Chemical Defense, Beijing 100191, China; mingzhongyuan2022@163.com (Z.M.); minjohng@126.com (M.Z.); zhangsxfhyjy@163.com (S.Z.); lxpbuct@163.com (X.L.); yanxiaoshan07@foxmail.com (X.Y.)

[2] Guangzhou Key Laboratory for New Energy and Green Catalysis, School of Chemistry and Chemical Engineering, Guangzhou University, Guangzhou 510006, China; 2112105072@e.gzhu.edu.cn (K.G.); liyu2021@e.gzhu.edu.cn (Y.L.); yfpeng@icost.ac.cn (Y.P.); 2112205096@e.gzhu.edu.cn (J.L.)

* Correspondence: zy291857261@126.com (Y.Z.); zqiao@gzhu.edu.cn (Z.Q.); liheguo1972@126.com (H.L.)

Catalogue

Lennard-Jones parameters of MOFs S3
TraPPE force field and Critical constants for the DES, N_2, and O_2, ... S4
One-hot encoding S5
Descriptors of the four types S6
Univariate analysis S7
Random Forest S8
Extreme Gradient Boosting S9
Light Gradient Boosting Machine S10
Categorical Boosting S11
Bayesian search S12
k-fold cross-validation S13
Evaluation index of Machine Learning S14
Parameters of Four Types of Machine Learning Models S15
Relative importance analysis S16
Shapley additive explanation S17
Uncertainty analysis method S18
Evaluation metrics for four ML algorithms S19
Evaluation of each ML algorithm by running the algorithm five times S20
Detailed information about the MACCS bits S21
Interaction force energy change S24
Simulation of CWAs using TraPPE force field S25
Discussion of simulation reliability for MOFs with open metal sites under generic force fields S27

Reference..S31

Lennard-Jones parameters of MOFs

Table S1 Lennard-Jones parameters of MOFs [1].

Atom	ε/kB [K]	σ [Å]	Atom	ε/kB [K]	σ [Å]	Atom	ε/kB [K]	σ [Å]
Ac	16.60	3.10	Ge	190.69	3.81	Po	163.52	4.20
Ag	18.11	2.80	Gd	4.53	3.00	Pr	5.03	3.21
Al	254.09	4.01	H	22.14	2.57	Pt	40.25	2.45
Am	7.04	3.01	Hf	36.23	2.80	Pu	8.05	3.05
Ar	93.08	3.45	Hg	193.71	2.41	Ra	203.27	3.28
As	155.47	3.77	Ho	3.52	3.04	Rb	20.13	3.67
At	142.89	4.23	I	170.57	4.01	Re	33.21	2.63
Au	19.62	2.93	In	301.39	3.98	Rh	26.67	2.61
B	90.57	3.64	Ir	36.73	2.53	Rn	124.78	4.25
Ba	183.15	3.30	K	17.61	3.40	Ru	28.18	2.64
Be	42.77	2.45	Kr	110.69	3.69	S	137.86	3.59
Bi	260.63	3.89	La	8.55	3.14	Sb	225.91	3.94
Bk	6.54	2.97	Li	12.58	2.18	Sc	9.56	2.94
Br	126.29	3.73	Lu	20.63	3.24	Se	146.42	3.75
C	52.83	3.43	Lr	5.53	2.88	Si	202.27	3.83
Ca	119.75	3.03	Md	5.53	2.92	Sm	4.03	3.14
Cd	114.72	2.54	Mg	55.85	2.69	Sn	285.28	3.91
Ce	6.54	3.17	Mn	6.54	2.64	Sr	118.24	3.24
Cf	6.54	2.95	Mo	28.18	2.72	Ta	40.75	2.82
Cl	114.21	3.52	N	34.72	3.26	Tb	3.52	3.07
Cm	6.54	2.96	Na	15.09	2.66	Tc	24.15	2.67
Co	7.04	2.56	Ne	21.13	2.66	Te	200.25	3.98
Cr	7.55	2.69	Nb	29.69	2.82	Th	13.08	3.03
Cu	2.52	3.11	Nd	5.03	3.18	Ti	8.55	2.83
Cs	22.64	4.02	No	5.53	2.89	TI	342.14	3.87
Dy	3.52	3.05	Ni	7.55	2.52	Tm	3.02	3.01
Eu	4.03	3.11	Np	9.56	3.05	U	11.07	3.02
Er	3.52	3.02	O	30.19	3.12	V	8.05	2.80
Es	6.04	2.94	Os	18.62	2.78	W	33.71	2.73
F	25.16	3.00	P	153.46	3.69	Xe	167.04	3.92
Fe	6.54	2.59	Pa	11.07	3.05	Y	36.23	2.98
Fm	6.04	2.93	Pb	333.59	3.83	Yb	114.72	2.99
Fr	25.16	4.37	Pd	24.15	2.58	Zn	62.39	2.46
Ga	208.81	3.90	Pm	4.53	3.16	Zr	34.72	2.78

TraPPE force field for the DES, N_2, and O_2

Table S2 TraPPE force field for the DES, N_2, and O_2 [2,3].

	ε(K)	σ(Å)	Charge(e)
DES			
S	199.00	3.58	-0.3
CH2	46.00	3.95	0.15
CH3	98.00	3.75	0
N_2			
N_N_2	36.00	3.31	-0.48
N_com	0	0	+0.96
O_2			
O_O_2	49.00	3.02	+0.11
O_com	0	0	-0.23

Site adjacent to CH_3, com_O2 and com_N2: the center-of-mass of O_2 and N_2, respectively.

Critical constants for CWAs and simulants

Table S3 Critical constants for DES.

Systems	Temperature (K)	Pressure	Acentric factor
DES	557.15	3961807.5	0.293553

Calculation of trade-off variables

The TSN[4] method was used to balance the numerical relationship between N and S by logarithmic calculation of S, so that the order of magnitude difference between the two is reduced, which could better reflect which variable is more significant. The calculation formula is as follows:

$$\mathrm{TSN} = N_i \cdot \ln\left(S_{i/\mathrm{N_2+O_2}}\right) \quad \mathrm{S}\,(1)$$

where i stands for the adsorbate: DES.

One-hot encoding

One-hot encoding[5] is a data pre-processing technique utilized to convert categorical variables into a binary vector representation. In the context of machine learning and data analysis, it is necessary to transform categorical variables into a digital form for algorithmic processing and analysis. One-hot encoding creates a distinct binary feature column for each unique categorical value, where the column

corresponding to the sample's categorical value is set to 1 and the other columns are set to 0. Consequently, each categorical value is uniquely represented as a binary vector consisting of 0s and 1s. By employing one-hot encoding, we can remove any inherent ordering relationships among categorical variables, enabling machine learning algorithms to better comprehend and interpret these features. Moreover, one-hot encoding can enhance model performance by circumventing erroneous numerical relationships that may arise when treating categorical variables as continuous variables.

In this study, we employed one-hot encoding to convert the presence or absence of each metal element and topology into their respective binary feature columns. These one-hot encoded features were utilized as inputs for the model, aiding in the improved learning and prediction of the properties or behaviors associated with the metal centers and topologies of the MOF. Fig. S5 illustrates each row representing an individual MOF, with the total number of columns equating to the count of distinct metal elements among the 15,335 CoRE-MOFs. When implementing one-hot encoding, the 'get_dummies' function within the Python Pandas library can be employed, providing a convenient and efficient approach to conduct one-hot encoding on the data.

	Cu	Ag	Co	••••••••	Eu
MOF1	1	0	0		1
MOF2	0	1	0		0
MOF3	0	0	1		0
MOF4	0	0	0		1
MOF5	0	0	0		0
⋮					
MOFn	1	1	0		1

Figure S1. Schematic diagram of one-hot encoding.

Descriptors of the four types

Table S4. Detailed information of descriptors for the four types.

	Full form	Computational method
**Structural descriptors**		
LCD	largest cavity diameter	
PLD	pore limiting diameter	

VSA	volumetric surface area	
ρ	density	
Ø	porosity	
**Energy descriptors**		
K	Henry coefficient	
Q_{st}^{0}	heat of adsorption	
**Chemical descriptors**		
Metal %	metal percentage	$\text{Metal percentage} = \frac{N_{metal}}{N_{Total}} \times 100\%$
C%	carbon percentage	$\text{Carbon percentage} = \frac{N_{carbon}}{N_{Total}} \times 100\%$
H%	hydrogen percentage	$\text{Hydrogen percentage} = \frac{N_{hydrogen}}{N_{Total}} \times 100\%$
O%	oxygen percentage	$\text{Oxygen percentage} = \frac{N_{oxygen}}{N_{Total}} \times 100\%$
N%	nitrogen percentage	$\text{Nitrogen percentage} = \frac{N_{nitrogen}}{N_{Total}} \times 100\%$
TDU	total degree of unsaturation	$TDU = \frac{N_C \times 2 + N_N + 2 - (N_H + N_F + N_{Cl} + N_{Br})}{2}$
DU	degree of unsaturation	$DU = \frac{\text{Total degree of unsaturation}}{N_C}$
**Charge descriptors**		
AMC	Average metal charge	$AMC = \frac{1}{N_{metal}} \sum_{i=1}^{N_{metal}} Charge_i$
MPC	Most positive charge	$MPC = \max(Charge_1, Charge_2, \ldots, Charge_n)$

N_i represents the number of atom i.

Univariate analysis

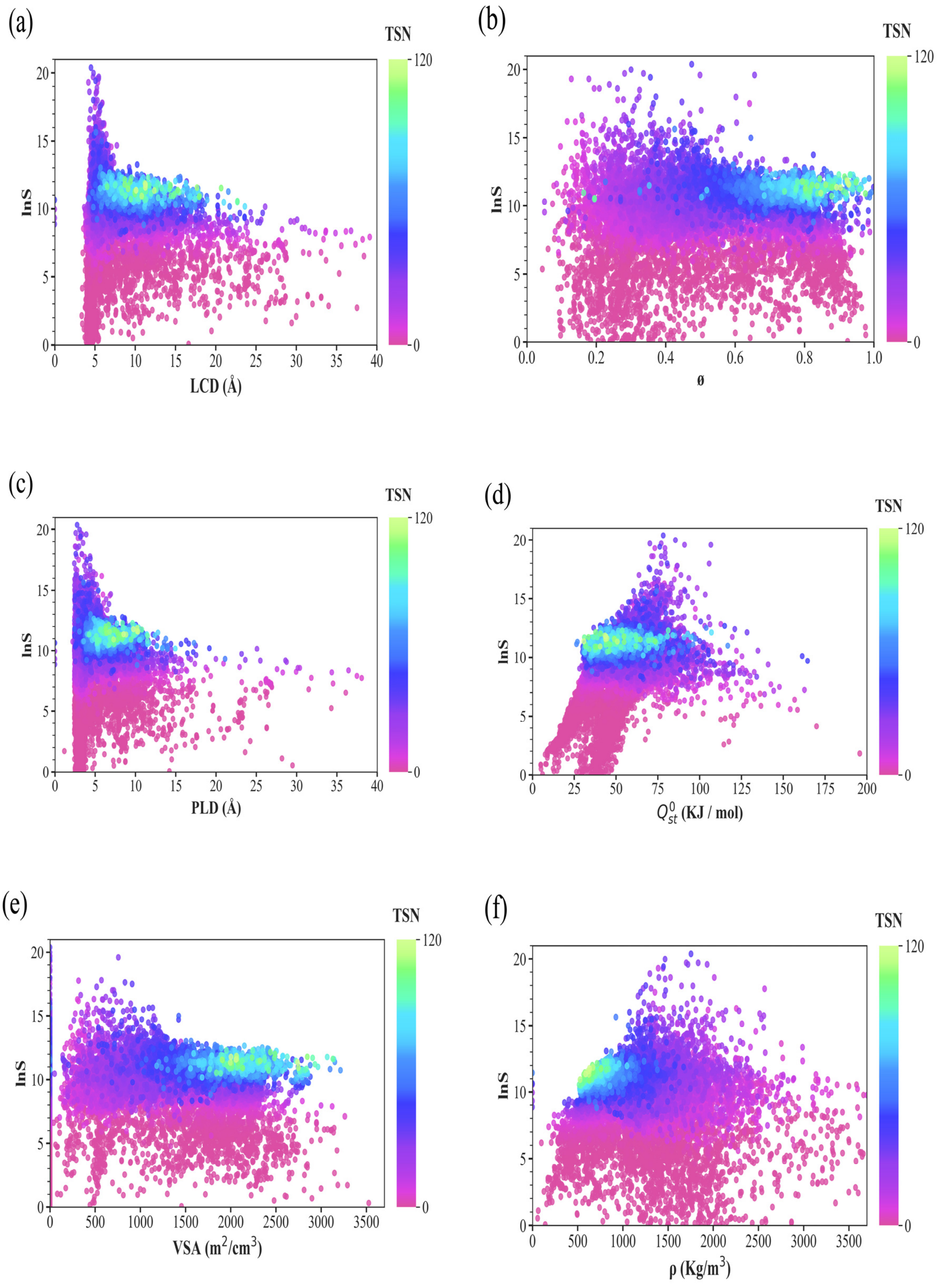

Figure S2. Descriptors-performance relationship between (a) LCD, lnS and TSN, (b) Ø,

lnS and TSN, (c) PLD, lnS and TSN, (d)Q^{0}_{st} ,lnS and TSN, (e)VSA , lnS and TSN, (f) ϱ, lnS and TSN.

Random Forest

Random Forest (RF) is an ensemble algorithm based on building a combinatorial decision tree model. In a random forest, each decision tree is built by predicting, growing, and segmenting input samples. When new samples are fed, they are evaluated by each decision tree with leaf nodes, and the predictions of each tree are aggregated, usually by averaging, to obtain the final model output, this comprehensive approach enables the random forest model to obtain high prediction accuracy and generalization performance, and the random forest with outstanding performance can be attributed to two key factors: randomness and the structure of the forest. It is embodied in a random sampling of the training subset, where random forests create different training subsets by randomly sampling from the original dataset, allowing each decision tree to train on a different sample set. This is a great help in mitigating the risk of overfitting and improving the generalization ability of the model. By combining the predictions of multiple decision trees, random forests can effectively reduce the errors inherent in a single decision tree and improve the robustness and stability of the overall model, so that we can accurately predict and interpret the phenomena and features related to a given data set.

As shown in Figure S1: a new training sample set is obtained by repeated random extraction of n from the initial training sample set. In this study, feature features are randomly selected and all features are trained; predictions for individual decision trees are obtained based on the sample extraction; and then the predictions for all decision trees are averaged to provide the final prediction. However, it can only generate predictions within the training set, which will lead to overfitting when modeling some noisy data.

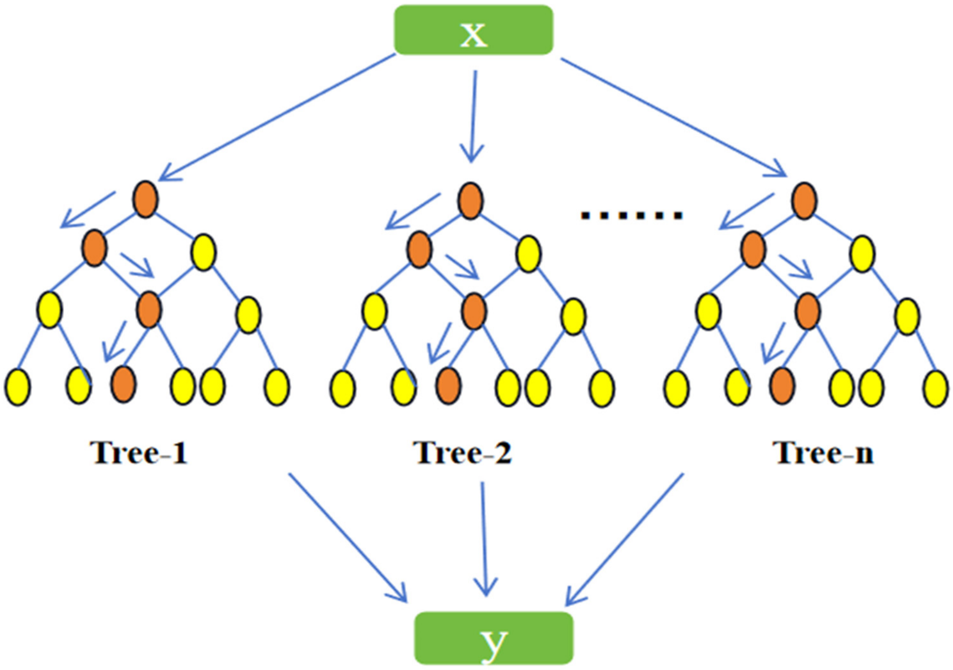

Figure S3. Random Forest regression.

Extreme Gradient Boosting

Extreme Gradient Boosting (XGB) has demonstrated superior performance on large-scale datasets and complex problems. XGB is an integrated algorithm based on gradient-enhanced decision trees that incrementally improves the predictive power of a model by iteratively adding decision trees. The core idea is to minimize the loss function by gradient descent and continuously fit the residuals of previous model predictions. XGB utilizes a decision tree as the base model and generates a new decision tree in each iteration to reduce the model's prediction error on the training data. By introducing a regularization term and using a second-order Taylor approximation to express the loss function, XGB effectively controls the complexity of the model and mitigates the overfitting problem. During the training process, XGB greedily constructs the decision tree and selects the optimal segmentation strategy by evaluating various segmentation points. As shown in Fig. S2, XGB has advantages including its ability to handle different data types and complex relationships, it can adapt to different data types and features, and it automatically learns the importance of features during model training. In addition, XGB maintains out strong robustness while maintaining a strong generalization ability by effectively dealing with noise in the data, such as missing values and outliers. In addition, choosing appropriate hyperparameters is crucial to optimize the performance of XGB. In conclusion, XGB is a powerful and flexible machine-learning algorithm with a wide range of applications and excellent performance. In our study, we use the XGB algorithm to solve specific problems and reveal underlying patterns and relationships in the data.

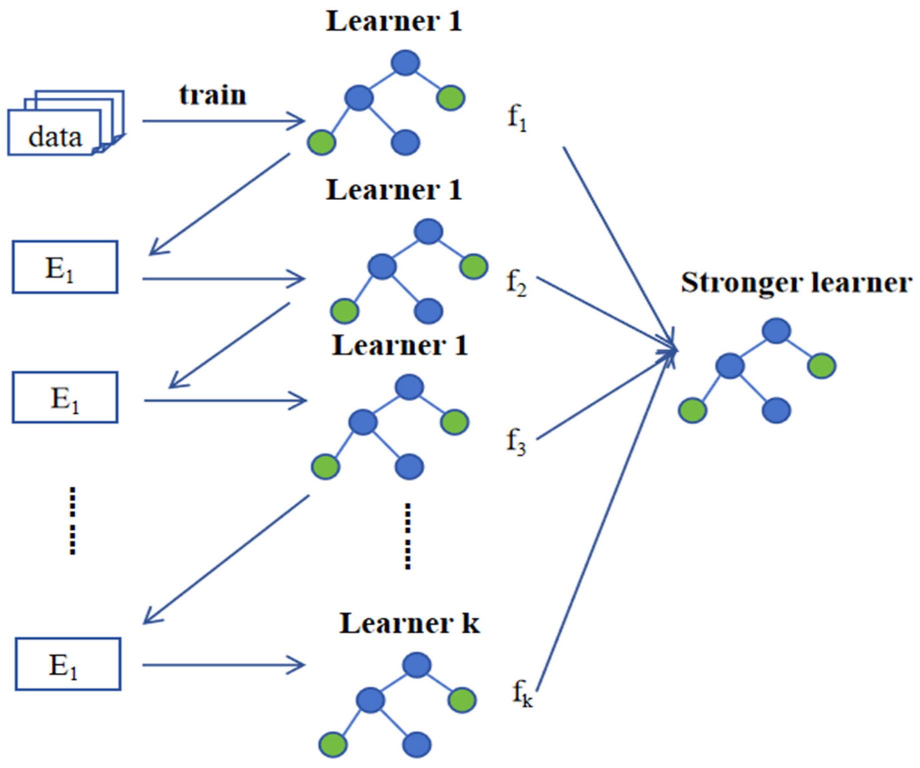

Figure S4. Extreme Gradient Boosting regression.

Light Gradient Boosting Machine

Light Gradient Boosting Machine (LGBM) is a framework that implements the GBDT (Gradient Boosting Decision Tree) algorithm, which can be trained in parallel with high efficiency, faster training speed, lower memory consumption, higher accuracy, and support for distributed and fast processing of massive data. One of the algorithms, in which the basic idea of the histogram algorithm is: first discretize the continuous floating-point eigenvalues into K integers, and at the same time, construct a histogram of width K. When traversing the data, based on the data, we can use the histogram as the basis for the algorithm. When traversing the data, statistics are accumulated in the histogram according to the discretized values as indexes, and when the data is traversed once, the histogram accumulates the required statistics and then traverses to find the optimal segmentation point based on the discrete values of the histogram.

LGBM adopts the Leaf-wise growth strategy, which finds a leaf with the largest splitting gain from all the current leaves each time, then splits it, and so on. Therefore, compared with Level-wise, the advantage of Leaf-wise is: that in the case of the same number of splits, Leaf-wise can reduce more errors and get better accuracy; the disadvantage of Leaf-wise is: that it may grow a deeper decision tree and produce overfitting. Therefore, LGBM adds a maximum depth limit on top of Leaf-wise to prevent overfitting while ensuring high efficiency.

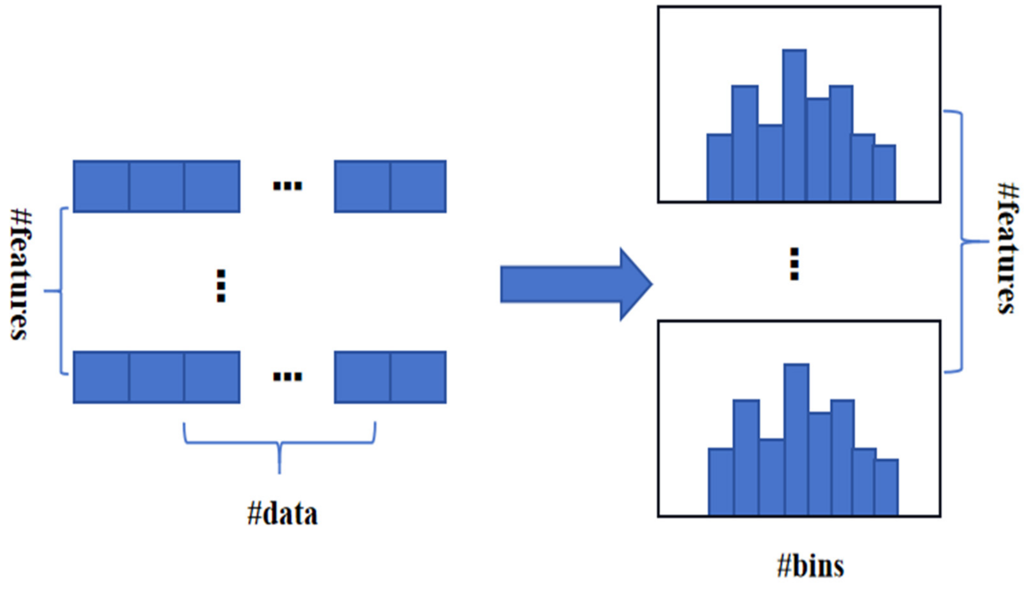

Figure S5. Light Gradient Boosting Machine regression.

Categorical Boosting (CatBoost)

The CatBoost algorithm is a highly optimized implementation within the Gradient Boosting on Regression Trees (GBRT) framework. CatBoost distinguishes itself by utilizing an oblivious tree structure, where all the internal decision nodes at the same level use the same splitting criterion, leading to balanced trees and efficient computation. This design not only simplifies the training process but also contributes to reducing the risk of overfitting compared to traditional GBRT implementations.

One of the key innovations of CatBoost is its ability to handle categorical features natively, without requiring manual preprocessing steps like one-hot encoding or label encoding. CatBoost uses an internal mechanism based on target-based encoding, where category values are transformed based on the distribution of the target variable, providing a more nuanced representation of categorical data. This allows the algorithm to efficiently and accurately capture relationships involving categorical variables, even when they have high cardinality.

Moreover, CatBoost introduces advanced techniques such as ordered boosting, which mitigates the prediction bias that can occur in traditional gradient boosting due to the sequential dependency between iterations. This is achieved by using permutations of the dataset to reduce target leakage, especially for small datasets.

In addition to its capability to handle categorical data, CatBoost is also known for having fewer hyperparameters to tune, which simplifies the model tuning process. It is highly accurate and robust to overfitting, particularly in situations where other boosting algorithms might struggle. Thanks to its efficient handling of large datasets, support for both categorical and numerical features, and built-in mechanisms for mitigating overfitting, CatBoost has become a powerful tool for both regression and classification tasks across various domains.

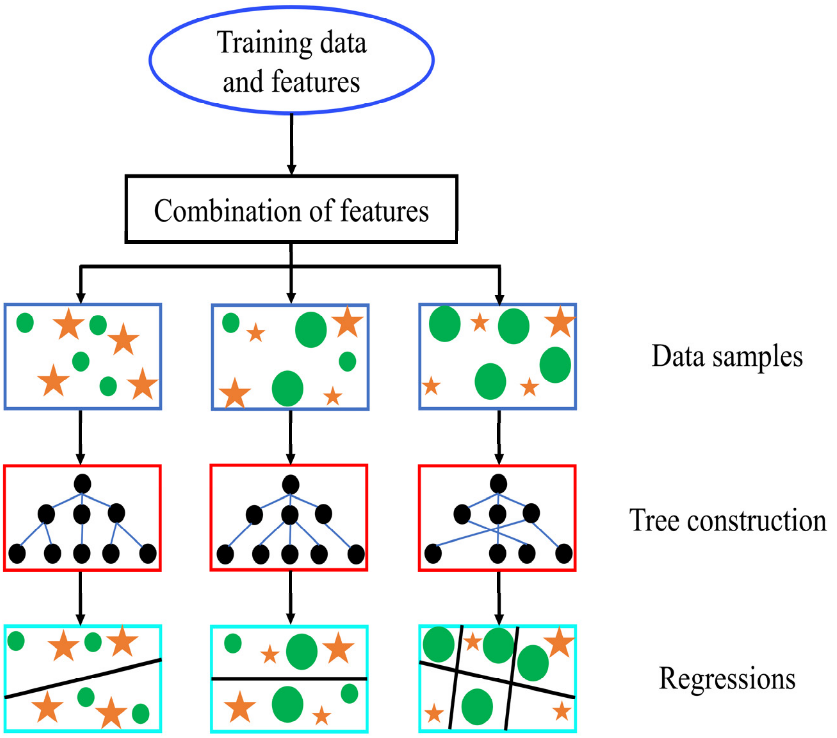

Figure S6. CatBoost Algorithm Flowchart.

Bayesian search

Bayesian search[6] is a Bayesian optimization-based search algorithm for finding the optimal solution or the best hyperparameter configuration for a problem. Its main role is to perform an efficient search in the parameter space to find the optimal configuration to optimize machine learning models or other optimization problems. the implementation of the Bayesian search algorithm is based on the idea of Bayesian optimization by building an agent model to approximate the underlying relationship of the objective function. It combines the methods of Bayesian statistics and Gaussian process regression to gradually collect data and update the proxy model to guide the search process, as shown in Fig. S10. Traditional grid search and random search require searching in a predefined parametric grid or random sampling space, which may lead to the dimensional explosion of the search space and thus inefficient search. In contrast, the Bayesian search algorithm approximates the objective function through a proxy model, which avoids a large number of random samples in the parameter space and can obtain better performance estimates on fewer sampling points, thus improving the search efficiency. Secondly, the Bayesian search algorithm can dynamically update the proxy model based on the existing sampling points, and thus select the next sampling point more intelligently. The traditional grid search and random search do not make use of the information already searched. this adaptive nature of Bayesian search can gradually adjust the search strategy during the search process and better adapt to the characteristics of the parameter space. In addition, the Bayesian search algorithm models the objective function through an agent model and takes into account the effects of uncertainty and noise. This makes the algorithm robust to the noise and uncertainty in the objective function and better able to cope with the challenges in real-world problems. It can be seen that the Bayesian search algorithm has significant advantages over the traditional grid search and random search in terms of search efficiency, adaptivity, and robustness. This makes it one of the important methods for hyperparametric optimization and machine learning model tuning.

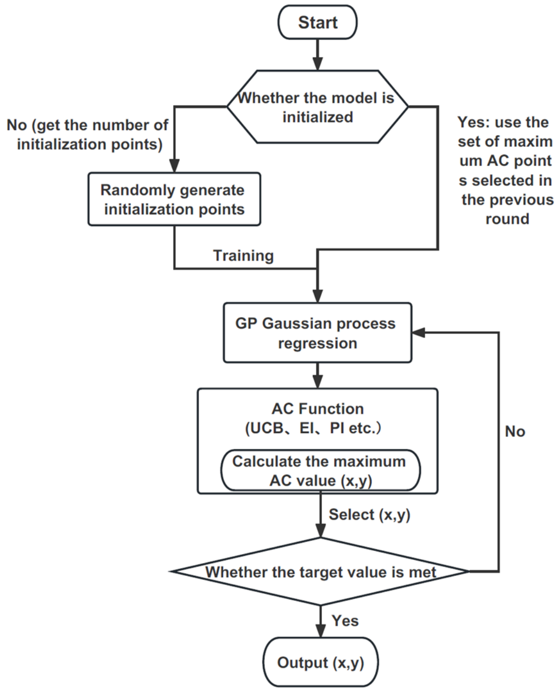

Figure S7. The schematic diagram of Bayesian optimization.

k-fold Cross Validation

k-fold cross-validation[7] is a commonly used model evaluation method to assess the performance and generalization ability of machine learning models. In *k*-fold cross-validation, the original data set is randomly divided into *k* subsets of the approximate size, where *k*-1 subsets are used as training data and the remaining 1 subset is used as validation data. The model is evaluated using a different validation set each time during these *k* training and validation sessions, as shown in Fig. S11. The specific procedure is as follows: The original data set is randomly divided into k subsets, each of which is called a fold. For each fold, it is used as the validation set, and the remaining *k*-1 folds are used as the training set. In each training session, the model is trained using the training set and evaluated on the validation set. The evaluation metrics of the model on each validation set, such as accuracy, precision, recall, etc., are recorded. Repeat steps 2 to 4 for a total of *k* times, using a different fold as the validation set each time. Finally, the *k*-validation results are averaged to obtain the final evaluation metrics. By using *k*-fold cross-validation, the performance of the model can be evaluated more accurately and the problem of over-reliance on a single validation set can be avoided. It can make full use of all samples in the dataset for training and validation, providing more stable and reliable model evaluation results. Also, *k*-weight cross-validation helps to evaluate the generalization ability of the model under different data distributions, thus better estimating the performance of the model on unknown data. The commonly used choices of *k* values are 5 and 10, but other suitable *k* values can be chosen depending on the size and characteristics of the data set. When performing k-weight cross-validation, care needs to be taken to ensure the randomness and representativeness of the data to avoid bias in the evaluation results due to uneven data distribution. The *k*-weight cross-validation is a widely used evaluation method, especially suitable for situations where the data set is small or needs to be fully utilized. It can provide researchers with a more accurate and reliable assessment of model performance, help select appropriate models and parameter configurations, and improve the generalization ability and robustness of machine learning models.

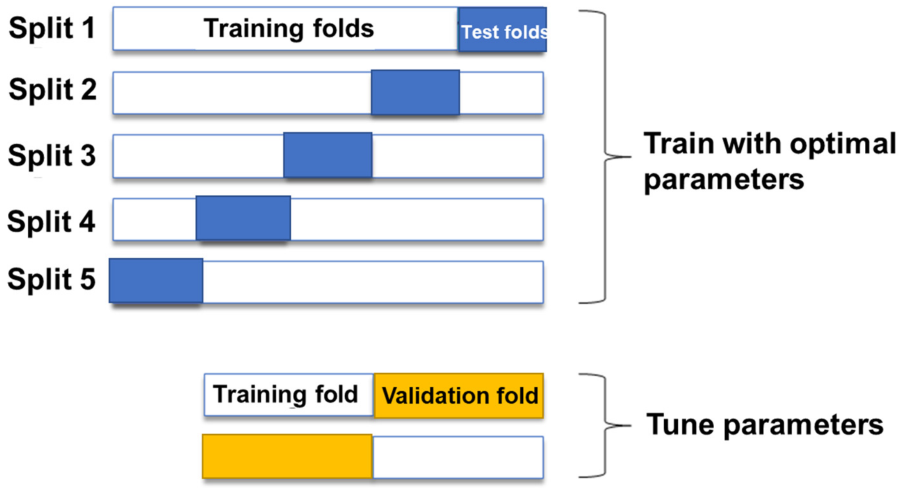

Figure S8. The diagram of *k*-fold cross-validation.

Evaluation index of Machine Learning

The evaluation criteria can be used to measure the difference between the simulated value and the predicted value. It has been widely used in various regression problems, such as mean absolute error (MAE), root mean square error (RMSE), and R. The calculation formulas are shown in formulas S (2)-S (4), where x_i is the simulated value of GCMC, y_i is the predicted value of machine learning, n is the number of MOFs, and $\overline{y}$ is the average value of the predicted value of machine learning.

$$\text{MAE}=\frac{1}{n}\sum_{i=1}^{n}|x_i-y_i| \tag{S2}$$

$$\text{RMSE}=\frac{1}{n}\sum_{i=1}^{n}(x_i-y_i)^2 \tag{S3}$$

$$R^2=1-\frac{\sum_{i=1}^{n}(x_i-y_i)^2}{\sum_{i=1}^{n}(y_i-\overline{y})^2} \tag{S4}$$

Table S5. Hyperparameters obtained by grid search with cross-validation.

ML algorithms	Function	Hyperparameters
RF	RandomForestRegressor	n_estimators=3000<br>max_depth=12<br>learning_rate=0.005<br>l2_leaf_reg=3.0

		verbose=200
LGBM	GBMRegressor	n_estimators=5000<br>max_depth=17<br>min_child_weight=2<br>subsample=0.8<br>min_samples_split=4<br>colsample_bytree=0.9<br>reg_alpha=0.3<br>reg_lambda=0.5<br>learning_rate=0.01
XGB	xgb.XGBRegressor	n_estimators=3000<br>max_depth=10<br>eta=0.005<br>min_child_weight=4<br>gamma=0.1<br>subsample=0.7<br>colsample_bytree=0.8<br>reg_lambda=0.8<br>reg_alpha=0.5
Catboost	CatBoostRegressor	iterations=3000<br>learning_rate=0.005<br>depth=12<br>l2_leaf_reg=3.0<br>verbose=200

Calculation of relative importance (RI)

The benefit of utilizing LGBM lies in its ability to provide clear and intuitive insights into the importance of features. By constructing boost trees, LGBM enables us to evaluate the relative importance (RI) of each feature, allowing us to identify the significant factors that have a substantial impact on the final model. The trained LGBM model automatically computes feature importance, which can be accessed through the 'feature importance' member variable. The RI of a feature is determined by its frequency of selection for splitting across the model's trees, with higher importance assigned to features selected more frequently. LGBM offers three methods to measure feature importance: 'weight,' which counts the number of times a feature is used for splitting data across all trees; 'gain,' which calculates the average gain achieved by utilizing a feature in the trees; and 'cover,' which assesses the average coverage of a feature in the trees. In this study, we adopt the 'weight' method in LGBM to determine a feature's RI, where a higher frequency of a feature being chosen for splitting indicates its greater importance within the trees. Understanding the feature importance in LGBM provides valuable insights into the significance of each feature in the model. This knowledge aids in identifying key variables that contribute significantly to the

model's performance. Incorporating this understanding enables informed decisions regarding feature selection, model refinement, and interpretation of results.

Shapley additive explanation

In this work, SHAP (Shapley Additive explanations) is used to explain the importance and role of different predictors in the analysis. As a game theory approach, SHAP interprets the predicted values of the model as the sum of the imputed values for each input feature, and when approximating the original model f for a particular input x, the explanation's attribution values ϕi for each feature *i* should sum up to the output *f(x)*, represented by equation S6:

$$f(x) = \phi_0(f) + \sum_{i=1}^{M} \phi_i\,(f, x) \qquad \text{(S5)}$$

Where the sum of the feature attributes $\varphi_i(f,x)$ matches the output *f(x)* of the original model, M is the total number of input features, ϕ_0 represents the expected value when all inputs are missing, and ϕi is a measure of the contribution of a given feature i to the prediction. According to game theory, the Shapley value is the only criterion that satisfies local accuracy, missing, and consistency. They are also very intuitive because they use the same units as the model output. SHAP value is the Shapley value of a conditional expectation function *f(x)*, which can be derived from equation S7:

$$\phi_i = \sum_{R\in\mathcal{R}} \frac{1}{M!}\left[f_x(P_i^R \cup i) - f_x(P_i^R)\right] \qquad \text{(S6)}$$

where R is the set of all feature orderings, P_i^R is the set of all features that come before feature i in ordering R, and M is the number of input features for the model. For tree-based models, our study utilizes the TreeExplainer algorithm developed by Lundberg et al[8], which adeptly calculates the SHAP values. The TreeExplainer assigns SHAP values to each individual sample within the dataset, providing a measure of the impact of each feature on the model's output[9]. Subsequently, these individual predictions are aggregated and visualized to offer a comprehensive, global interpretation of the model's behavior.

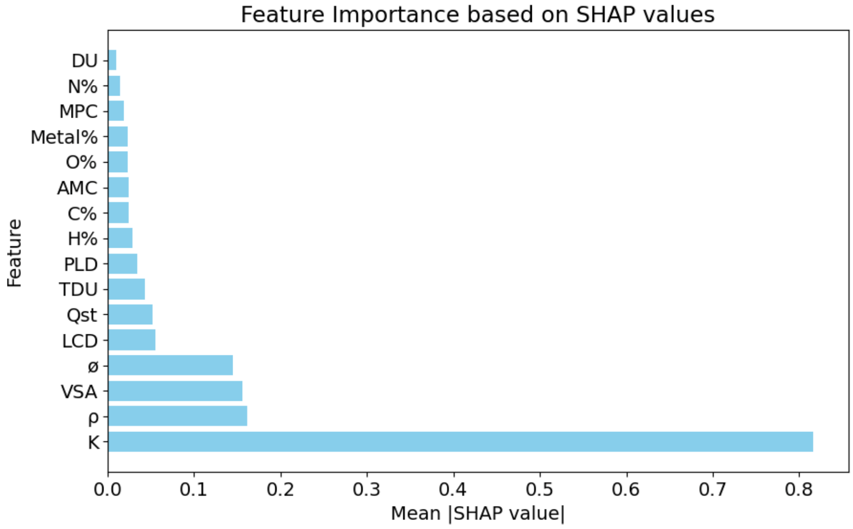

Figure S9. Feature Importance based on SHAP values.

Uncertainty Analysis Method

To evaluate the transferability of the machine learning model across different datasets, we performed an uncertainty analysis. This analysis aims to quantify the uncertainty of the model by making multiple predictions on the same dataset and measuring the variations in those predictions. The specific methods and steps are as follows:

First, the dataset underwent a series of preprocessing steps. To ensure the model's stability and accuracy when handling the data, a logarithmic transformation was applied to address potential issues related to extreme values or zero values. Following preprocessing, the dataset was split into training and testing sets. The model was trained on the training set using the LightGBM algorithm, with parameter tuning and cross-validation employed to ensure optimal performance. After training, the model was used to make predictions on the testing set to assess its performance on unseen data.

On the testing set, multiple iterations of predictions were performed. To quantify the model's uncertainty, 100 prediction iterations were conducted under the same dataset and model conditions. The model structure and input data were kept consistent across each iteration to ensure the uniformity of the prediction process. Each iteration produced a set of predictions, and by comparing the differences among these iterative predictions, the stability of the model's predictions on the same dataset could be observed.

After obtaining the predictions from all iterations, the standard deviation of the predictions was calculated. The standard deviation serves as a metric for the dispersion of the predictions, representing the model's volatility. A larger standard deviation indicates a higher level of uncertainty in the model's predictions for that dataset. The standard deviation for each test sample was computed based on the 100

predictions, and the average standard deviation across all samples was calculated to determine the overall uncertainty value for the dataset. This uncertainty value is used to quantify the model's transferability and stability across different datasets. Through uncertainty analysis, a more comprehensive assessment of the model's performance in new environments can be obtained, providing a deeper understanding of its generalization capability when combined with other performance metrics.The model uncertainty for the new data is 1.268702639131082e-15, and the model uncertainty for the old data is 2.7649857758040184e-15.

Table S6. Evaluation metrics for four ML algorithms.

Performance Indicators	Machine Learning Methods		Average		
			R^2	MAE	RMSE
N_{DES}	training	RF	0.937	0.089	0.574
		XGB	0.966	0.068	0.370
		CatBoost	0.969	0.065	0.342
		LGBM	0.990	0.036	0.188
	test	RF	0.907	0.107	0.663
		XGB	0.930	0.090	0.542
		CatBoost	0.934	0.088	0.531
		LGBM	0.941	0.080	0.500
S	training	RF	0.876	0.677	10596125
		XGB	-143.26	34.148	2.59e+19
		CatBoost	0.937	0.501	10415215
		LGBM	0.981	0.278	8639312
	test	RF	0.844	0.767	9989694
		XGB	-142.96	34.148	2.59e+19
		CatBoost	0.876	0.664	9985486
		LGBM	0.886	0.615	9973585
TSN	training	RF	0.951	0.189	7.980
		XGB	0.984	0.107	3.858
		CatBoost	0.972	0.141	5.021
		LGBM	0.991	0.077	2.905
	test	RF	0.929	0.225	8.785
		XGB	0.953	0.178	6.585
		CatBoost	0.947	0.188	6.917
		LGBM	0.953	0.170	6.435

Table S7 Evaluation of each ML algorithm by running the algorithm five times.

ML Algorithm	CWAs

			DES				
			1	2	3	4	5
XGB	Train	R^2	0.984	0.984	0.984	0.984	0.984
		MAE	0.108	0.107	0.107	0.107	0.108
		RMSE	3.880	3.819	3.869	3.869	3.856
	Test	R^2	0.952	0.946	0.949	0.948	0.956
		MAE	0.179	0.182	0.179	0.180	0.170
		RMSE	6.528	6.795	6.294	6.602	6.707
RF	Train	R^2	0.950	0.952	0.952	0.952	0.951
		MAE	0.190	0.188	0.189	0.188	0.190
		RMSE	8.050	7.925	8.020	8.003	7.903
	Test	R^2	0.931	0.924	0.927	0.925	0.936
		MAE	0.229	0.231	0.224	0.229	0.216
		RMSE	8.970	9.090	8.253	8.610	9.004
LGBM	Train	R^2	0.992	0.992	0.992	0.992	0.992
		MAE	0.078	0.077	0.077	0.077	0.079
		RMSE	2.923	2.891	2.866	2.905	2.941
	Test	R^2	0.955	0.950	0.952	0.952	0.957
		MAE	0.171	0.173	0.171	0.172	0.165
		RMSE	6.382	6.531	6.152	6.465	6.645
CatBoost	Train	R^2	0.972	0.974	0.974	0.972	0.973
		MAE	0.142	0.139	0.140	0.142	0.142
		RMSE	5.126	4.925	4.965	5.050	5.038
	Test	R^2	0.948	0.945	0.947	0.945	0.951
		MAE	0.192	0.190	0.186	0.191	0.182
		RMSE	7.000	6.991	6.483	6.962	7.151

Detailed Explanation of Frequency Threshold Selection and Its Rationality

In this study, to identify molecular fingerprint features closely associated with the adsorption performance of MOFs, we converted all material samples into molecular fingerprints and statistically analyzed the fingerprint frequencies across different performance categories (Top materials, Middle materials, and Bottom materials). Given the disparity in sample sizes and feature distributions among these categories, specific frequency thresholds were set to ensure the reliability and robustness of the screening results. The rationale and appropriateness of these thresholds are elaborated

as follows.

(1) Defining and Excluding Ordinary Bits: Fingerprint features that appeared with a frequency exceeding 80% across all three categories were defined as ordinary bits. These features, due to their ubiquitous presence, lacked the discriminatory power to distinguish materials with varying adsorption performances. As such, ordinary bits were excluded from further analysis to prevent interference in the identification of performance-specific features. This step aligns with standard practices in high-throughput data screening, where overly frequent or rare features are typically treated as noise and excluded from further consideration.

(2) 50% Threshold for Top Materials: Given the small sample size of Top materials (247 samples, ~1% of the total), a 50% threshold was chosen to retain fingerprints appearing in more than 50% of this group. This threshold balances sensitivity and stability for identifying meaningful features in limited datasets.

(3) 70% Threshold for Bottom Materials: For Bottom materials (7,653 samples, >50% of the total), a 70% threshold was applied to select fingerprints with high prevalence. This ensures that the retained features are representative while filtering out low-frequency noise.

(4) Balancing Threshold Selection: By setting thresholds of 50% for Top materials and 70% for Bottom materials, this study achieves a balance between noise filtering and feature identification. This approach accommodates sample size differences while ensuring reliable screening results.

Table S8. Detailed information about the bits.

Bit	TOP(%)	Bottom(%)	Overall(%)	SMART	Structure	Description
**66**	14.98	31.11	29.35	('[#6]~[#6](~[#6])(~[#6])~*', 0)	CC(C)(C)A	A carbon atom

						structure connected to at least 3 carbon atoms and 1 arbitrary atom.
**79**	10.12	27.98	26.69	('[#7]~*~*~[#7]', 0)	NAAN	Two nitrogen atoms connected by two arbitrary atoms.
**120**	10.12	28.96	27.77	('[!#6;R]', 1)	Heterocyclic atom > 1	A non-carbon atom in a ring structure.
**121**	23.89	41.80	40.47	('[#7;R]', 0)	N Heterocycle	A nitrogen atom in a ring structure
**122**	23.08	39.46	38.17	('*~[#7](~*)~*', 0)	AN(A)A	A nitrogen atom connected to three arbitrary atoms.
**124**	63.56	61.18	64.23	('[!#6;!#1]~[!#6;!#1]', 0)	QQ	Two non-carbon, non-hydrogen atoms connected by any type of bond.

**125**	14.17	29.44	25.57	‘?’,0	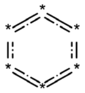	The number of aromatic ring structure is more than 1.
**126**	68.83	58.26	63.55	('*!@[#8]!@*', 0)	A!O!A	An oxygen atom connected to two arbitrary atoms via non-ring bonds.
**131**	14.17	29.26	25.40	('[!#6;!#1;!H0]', 1)	QH > 1	One atom except C and H connected with H atom is more than 1.
**138**	24.29	42.02	40.70	('[!#6;!#1]~[CH2]~*', 1)	QCH2A>1	A non-carbon, non-hydrogen atom connected to a CH_2 group and an arbitrary atom.
**146**	70.85	56.46	62.31	('[#8]', 2)	O > 2	The number of oxygen is more than 2.
**158**	63.97	63.75	66.60	('[#6]-[#7]', 0)	C-N	A carbon atom

						connected to a nitrogen atom by a single bond.
**162**	32.79	52.25	49.25	('a', 0)	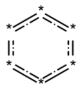	Aromatic is existed in structure.
**163**	69.64	59.90	66.00	('*1~*~*~*~*~*~1', 0)	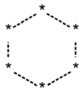	The structure is existing 6-membered ring.
**164**	74.90	58.36	65.18	('[#8]', 0)	O	Oxygen atom.

*represents any atom, ~ represents any bond.

Interaction force energy change

To explore the interactions between the gases and MOFs during the adsorption process, we analyzed the adsorbate-adsorbent interactions in two top-performing MOFs during GCMC simulation. The van der Waals dominates the interaction energy between adsorbate-adsorbent (75% of total energy), indicating that van der Waals interaction governs the adsorption process of CWAs on MOFs. This is because the MOFs can provide suitable pore spaces for the adsorption of CWAs, resulting in an increased contribution of van der Waals forces during the adsorption process[10,11].

Simulation of CWAs using TraPPE force field

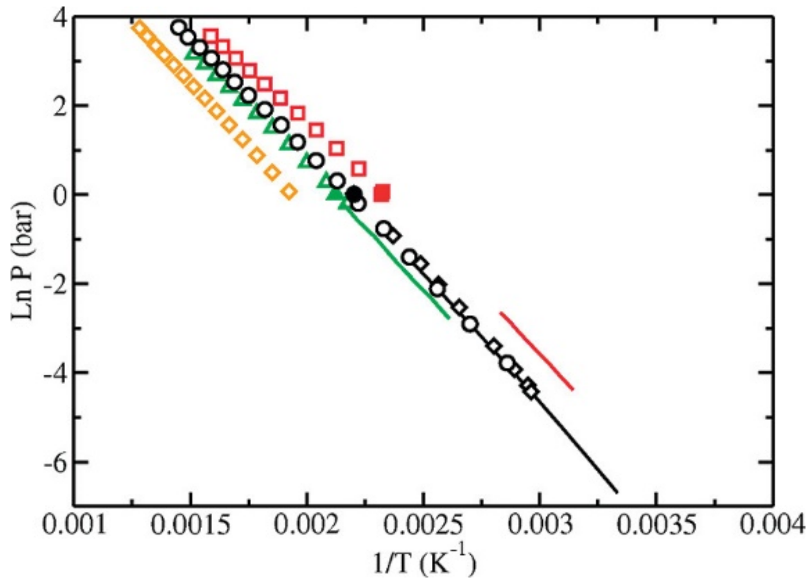

Figure S10. Clausius−Clapeyron plot for DMMP (circle), DMMP-VN (diamond), sarin (square), and soman (triangle). Line reperesents the experimental vapor pressure of DMMP (black), sarin (red), and soman (green). Filled symbols represent experimental boiling points. Black diamonds correspond to a recently published set of experimental vapor pressure data for DMMP[3].

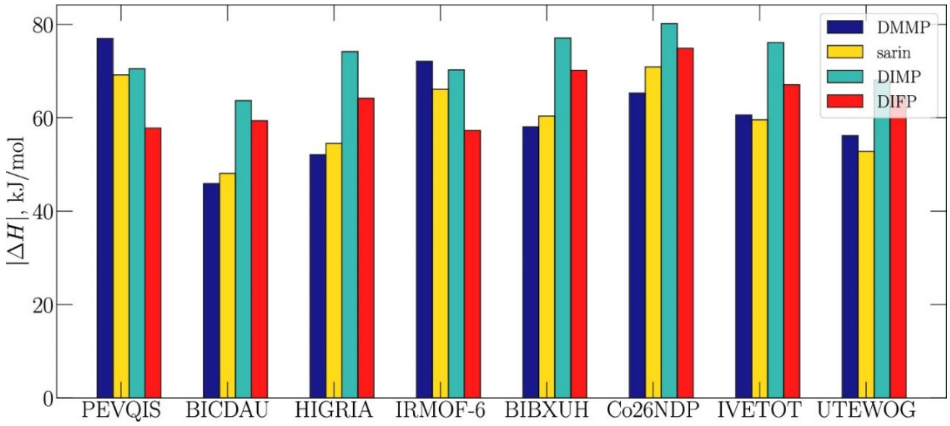

Figure S11. Absolute value of the enthalpy of adsorption at infinite dilution ΔH of sarin and simulants in MOFs at 298 K[12].

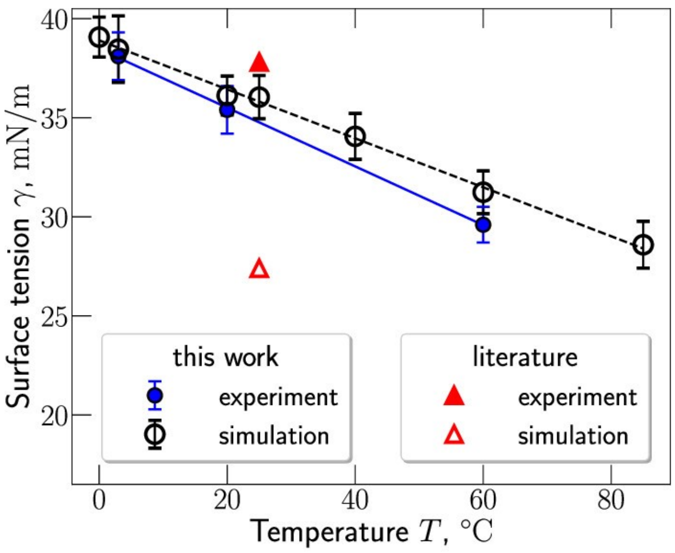

Figure S12. Surface tension of DMMP[13].

Discussion of simulation reliability for MOFs with open metal sites under generic force fields

Siegel group employed molecular simulations using the Universal force field to investigate the CH_4 and CO_2 adsorption performance of HKUST-1 materials with open metal sites, and the qualitative agreement between calculated and experimental adsorption isotherms demonstrated the accuracy of force field[9]. Additionally, Farha et al. successfully utilized the Universal force field to simulate the benzene adsorption isotherms of Mg-MOF-74, a MOF featuring open metal sites, and compared them to experimental data, yielding excellent agreement[10]. These studies support the suitability of the Universal force field for simulating experiments on MOFs containing open metal sites.

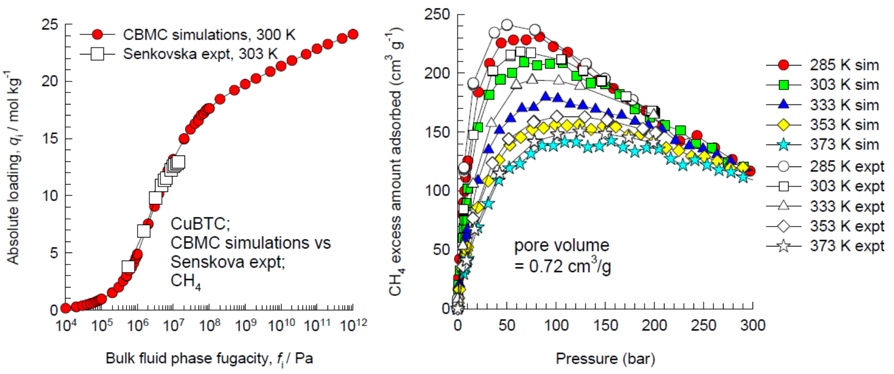

(a) CuBTC for CH_4 (b) CuBTC for CH_4

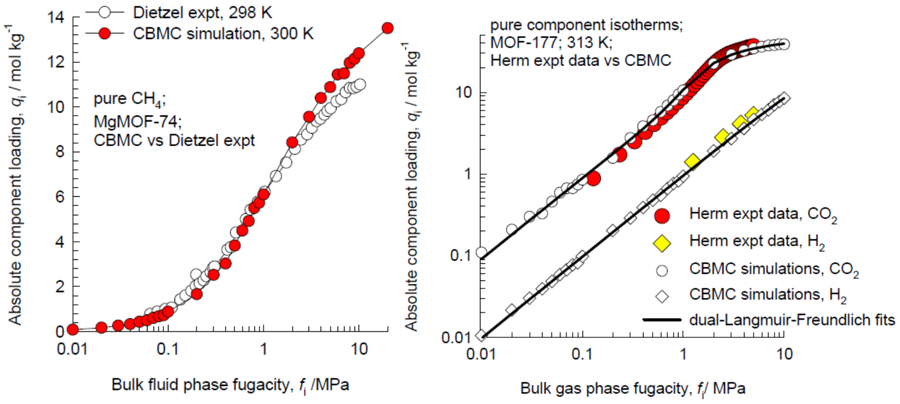

(c) Mg-MOF-74 for CH_4 (d) MOF-177 for CO_2 and H_2

Figure S13. Comparison between experimental and simulated adsorption isotherms[14].

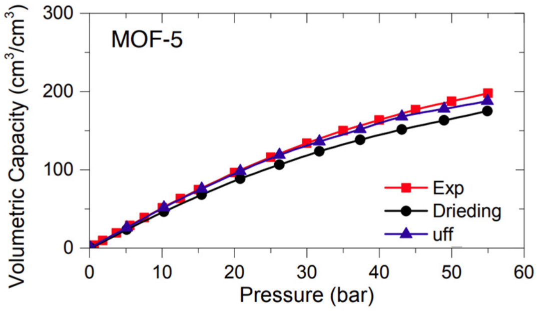

Figure S14. Comparison of the experimental CH_4 isotherms with isotherms calculated[15].

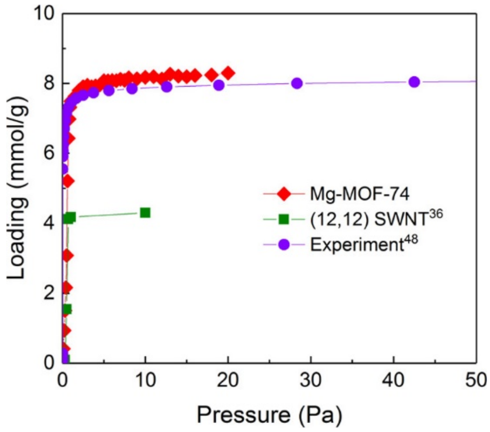

Figure S15. Comparison of simulated adsorption isotherms in Mg-MOF-74 with the experimental one[16].

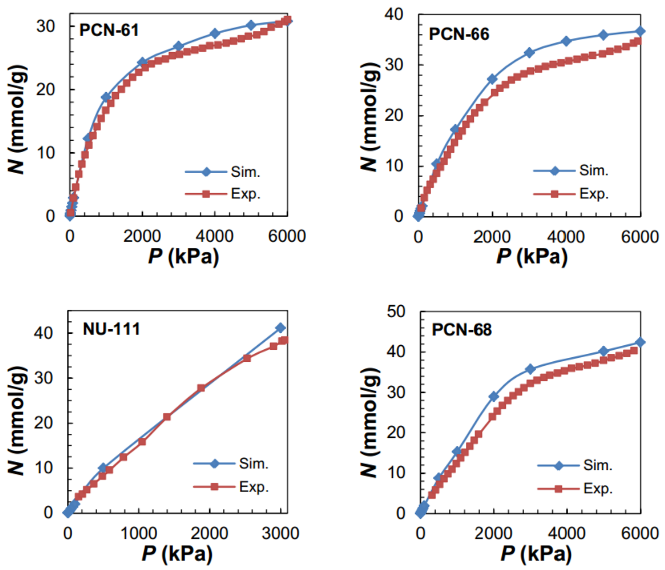

(a) CO_2 isotherms

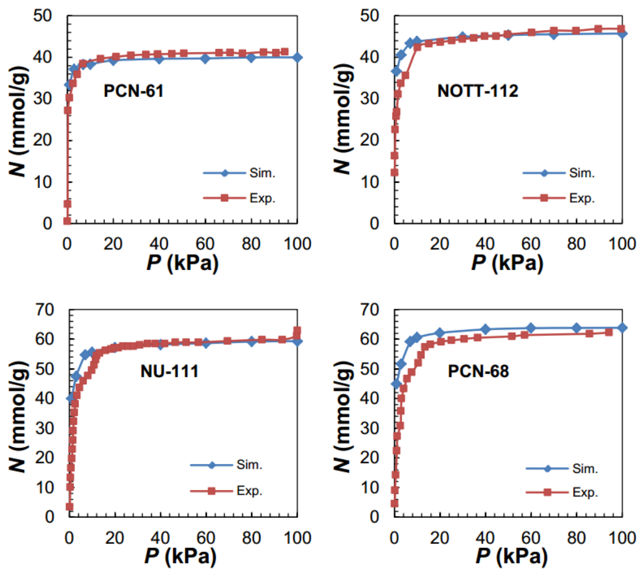

(b) N_2 isotherms

Figure S16. Adsorption isotherms of CO_2 and N_2 at 298 K[17].

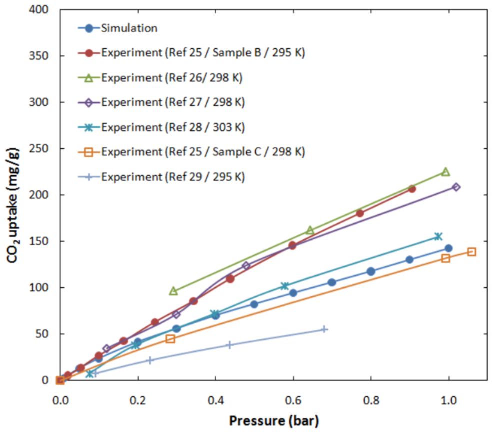

(a) HKUST-1

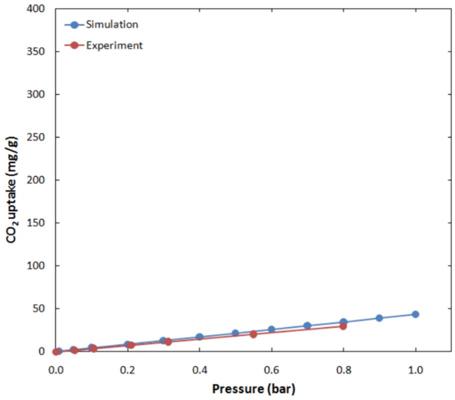

(b) IRMOF-1

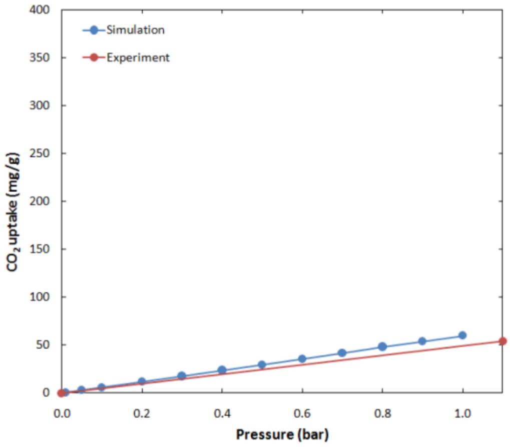

(c) IRMOF-3

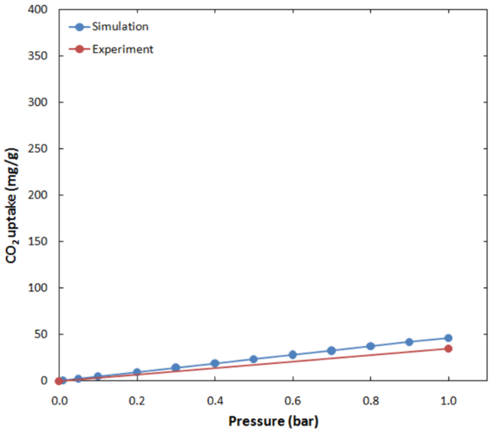

(d) MOF-177

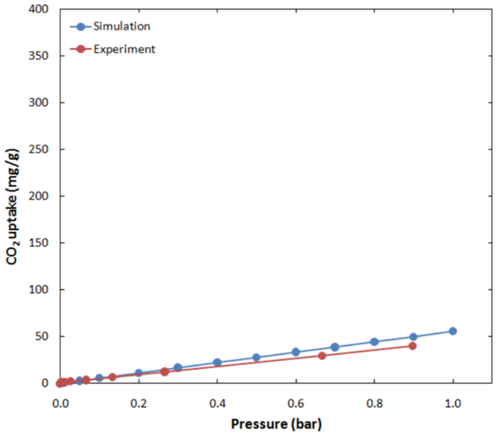

(e) ZIF-8

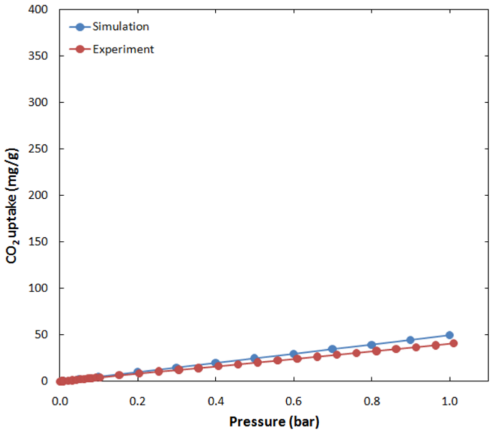

(f) UMCM-1

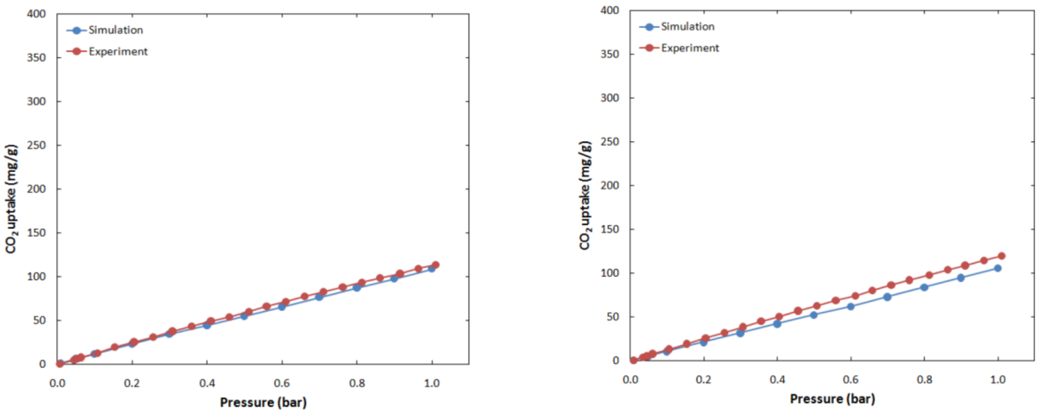

(g) UMCM-150 (h) UMCM-150(N)2

Figure S17. CO_2 isotherms in various MOFs[18].

References

1. Rappe, A.K.; Casewit, C.J.; Colwell, K.S.; Goddard, W.A.; Skiff, W.M. UFF, a Full Periodic Table Force Field for Molecular Mechanics and Molecular Dynamics Simulations. *J. Am. Chem. Soc.* **1992**, *114*, 10024–10035, doi:10.1021/ja00051a040.
2. Matito-Martos, I.; Moghadam, P.Z.; Colombo, V.; Navarro, J.A.R.; Calero, S.; Fairen-Jimenez, D. Discovery of an Optimal Porous Crystalline Material for the Capture of Chemical Warfare Agents. *Chem. Mater.* 4571–4579, doi:10.1021/acs.chemmater.8b00843.
3. Sokkalingam, N.; Kamath, G.; Coscione, M.; Potoff, J.J. Extension of the Transferable Potentials for Phase Equilibria Force Field to Dimethylmethyl Phosphonate, Sarin, and Soman. *J. Phys. Chem. B* **2009**, *113*, 10292–10297, doi:10.1021/jp903110e.
4. Shah, M.S.; Tsapatsis, M.; Siepmann, J.I. Identifying Optimal Zeolitic Sorbents for Sweetening of Highly Sour Natural Gas. *Angew Chem Int Ed*

2016, *55*, 5938–5942, doi:10.1002/anie.201600612.

5. Lam Pham, T.; Kino, H.; Terakura, K.; Miyake, T.; Tsuda, K.; Takigawa, I.; Chi Dam, H. Machine Learning Reveals Orbital Interaction in Materials. *Sci. Technol. Adv. Mater.* **2017**, *18*, 756–765, doi:10.1080/14686996.2017.1378060.
6. Nguyen, V. Bayesian Optimization for Accelerating Hyper-Parameter Tuning. In Proceedings of the 2019 IEEE Second International Conference on Artificial Intelligence and Knowledge Engineering (AIKE); IEEE: Sardinia, Italy, June 2019; pp. 302–305.
7. Yan, Y.; Shi, Z.; Li, H.; Li, L.; Yang, X.; Li, S.; Liang, H.; Qiao, Z. Machine Learning and In-Silico Screening of Metal–Organic Frameworks for O2/N2 Dynamic Adsorption and Separation. *Chem. Eng. J.* **2022**, *427*, 131604, doi:10.1016/j.cej.2021.131604.
8. Lundberg, S.M.; Erion, G.; Chen, H.; DeGrave, A.; Prutkin, J.M.; Nair, B.; Katz, R.; Himmelfarb, J.; Bansal, N.; Lee, S.-I. From Local Explanations to Global Understanding with Explainable AI for Trees. *Nat Mach Intell* **2020**, *2*, 56–67, doi:10.1038/s42256-019-0138-9.
9. Fujimoto, K.; Kojadinovic, I.; Marichal, J.-L. Axiomatic Characterizations of Probabilistic and Cardinal-Probabilistic Interaction Indices. *Games Econ. Behav.* **2006**, *55*, 72–99, doi:10.1016/j.geb.2005.03.002.
10. Louw, K.I.; Bradshaw-Hajek, B.H.; Hill, J.M. Interaction of Ferric Ions with Europium Metal Organic Framework and Application to Mineral

Processing Sensing. *Philosophical Magazine* **2022**, *102*, 1231–1246, doi:10.1080/14786435.2022.2061066.

11. Hermann, J.; DiStasio, R.A.; Tkatchenko, A. First-Principles Models for van Der Waals Interactions in Molecules and Materials: Concepts, Theory, and Applications. *Chem. Rev.* **2017**, *117*, 4714–4758, doi:10.1021/acs.chemrev.6b00446.
12. Emelianova, A.; Reed, A.; Basharova, E.A.; Kolesnikov, A.L.; Gor, G.Y. Closer Look at Adsorption of Sarin and Simulants on Metal–Organic Frameworks. *ACS Appl. Mater. Interfaces* **2023**, *15*, 18559–18567, doi:10.1021/acsami.3c02713.
13. Ivanova, E.V.; Vasudevan, A.; Senyurt, E.I.; Schoenitz, M.; Khalizov, A.F.; Dreizin, E.L.; Gor, G.Y. Surface Tension of Organophosphorus Compounds: Sarin and Its Surrogates. *Langmuir* **2023**, *39*, 5569–5578, doi:10.1021/acs.langmuir.3c00460.
14. Krishna, R.; Van Baten, J.M. In Silico Screening of Metal–Organic Frameworks in Separation Applications. *Phys. Chem. Chem. Phys.* **2011**, *13*, 10593, doi:10.1039/c1cp20282k.
15. Koh, H.S.; Rana, M.K.; Wong-Foy, A.G.; Siegel, D.J. Predicting Methane Storage in Open-Metal-Site Metal–Organic Frameworks. *J. Phys. Chem. C* **2015**, *119*, 13451–13458, doi:10.1021/acs.jpcc.5b02768.
16. Liu, A.; Peng, X.; Jin, Q.; Jain, S.K.; Vicent-Luna, J.M.; Calero, S.; Zhao, D. Adsorption and Diffusion of Benzene in Mg-MOF-74 with Open Metal

Sites. *ACS Appl. Mater. Interfaces* **2019**, *11*, 4686–4700, doi:10.1021/acsami.8b20447.

17. Zhang, K.; Nalaparaju, A.; Jiang, J. CO_2 Capture in **Rht** Metal–Organic Frameworks: Multiscale Modeling from Molecular Simulation to Breakthrough Prediction. *J. Mater. Chem. A* **2015**, *3*, 16327–16336, doi:10.1039/C5TA01866H.
18. Yazaydın, A.Ö.; Snurr, R.Q.; Park, T.-H.; Koh, K.; Liu, J.; LeVan, M.D.; Benin, A.I.; Jakubczak, P.; Lanuza, M.; Galloway, D.B.; et al. Screening of Metal−organic Frameworks for Carbon Dioxide Capture from Flue Gas Using a Combined Experimental and Modeling Approach. *J. Am. Chem. Soc.* **2009**, *131*, 18198–18199, doi:10.1021/ja9057234.
